# Plasiatine, an Unprecedented Indole–Phenylpropanoid Hybrid from *Plantago asiatica* as a Potent Activator of the Nonreceptor Protein Tyrosine Phosphatase Shp2

**DOI:** 10.1038/srep24945

**Published:** 2016-04-22

**Authors:** Zhong-Hua Gao, Yi-Ming Shi, Zhe Qiang, Xia Wang, Shan-Zhai Shang, Yan Yang, Bao-Wen Du, Hui-Pan Peng, Xu Ji, Honglin Li, Fei Wang, Wei-Lie Xiao

**Affiliations:** 1 State Key Laboratory of Phytochemistry and Plant Resources in West China, Kunming Institute of Botany, Chinese Academy of Sciences, Kunming, 650201, P. R. China; 2 Guangdong Provincial Academy of Chinese Medical Sciences, Guangzhou, 510006, P. R. China; 3 Chengdu Institute of Biology, Chinese Academy of Sciences, Chengdu, 610041, P. R. China; 4 Shanghai Key Laboratory of New Drug Design, School of Pharmacy, East China University of Science and Technology, Shanghai, 200237, P. R. China

## Abstract

Plasiatine (**1**), isolated from the seeds of *Plantago asiatica*, is an unprecedented indole analogue linked to a phenylpropanoid moiety via a carbon bond that builds up a novel heteromeric construction with a C_19_N_2_ scaffold. Its structure was determined by spectroscopic data and computational evidence. Notably, experimental assay demonstrated that **1** significantly enhanced the activity of the nonreceptor protein tyrosine phosphatase Shp2 *in vitro* in a concentration-dependent manner with an EC_50_ value of 0.97 *μ*M, and activated phosphorylation of ERK, a known target of Shp2. Moreover, plasiatine (**1**) promoted hepatocellular HepG2 cells migration. Molecular docking suggested that plasiatine (**1**) binds to the catalytic cleft of Shp2. These results identified plasiatine (**1**) as the first small molecule Shp2 activator, and it warrants further investigation as a novel pharmaceutical tool to study the function of Shp2 in tumorigenesis.

The Src homology 2-containing phosphotyrosine phosphatase 2 (Shp2) encoded by *PTPN11* gene is a nonreceptor protein tyrosine phosphatase^1^. This phosphatase is a pivotal transducer of growth factors and cytokines, and is a critical intracellular regulator in mediating cell proliferation, differentiation, adhesion, migration, and apoptosis^2^,^3^. Somatic activating mutations in Shp2 have been demonstrated to be linked to hematologic disorders (e.g. juvenile myelomonocytic leukemia, myelodysplastic syndromes, acute myeloid leukemia, and acute lymphoid leukemia)^4^, and several types of solid tumors (e.g. lung adenocarcinoma, colon cancer, neuroblastoma, glioblastoma, melanoma, and breast carcinoma)^5^. In the last ten years, enormous efforts directed toward the discovery of specific Shp2 inhibitors as potential therapeutic agents for hematologic malignancies and solid tumors have been continuing in several research groups^6^,^7^,^8^,^9^,^10^,^11^. However, a paradoxical function demonstrated recently is that the appearance of inflammatory signaling through the Stat3 pathway and hepatic inflammation/necrosis is promoted by hepatocyte-specific deletion or inactivation of Shp2, leading to regenerative hyperplasia and development of cancers^12^,^13^. Therefore, the function of Shp2 in tumorigensis may be tissue-specific, and it is also important to discover Shp2 activators to dissect Shp2-meidated signaling pathways. However, such explorations are still lacking.

*Plantago asiatica*, a species of the *Plantago* genus of the Plantaginaceae family distributed worldwide, is a famous traditional Chinese medicine (TCM)^14^. Its seed is used as diuretic, detoxification, expectorant, and laxative; and its whole plant is used for the treatment of fever, sputum, and inflammatory^15^,^16^,^17^. The secondary metabolites of *P. asiatica* are characterized by phenylethanoid glycosides^18^,^19^,^20^,^21^ and other phenolic compounds^22^. In order to obtain structurally interesting and biologically active secondary metabolites, our preliminary study for chemical constituents of the seeds of *P. asiatica* provided a series of simple indole alkaloids, plasiaticines A–D, (+)-(*R*)-3-cyanomethyl-3-hydroxyoxindole, and indolyl-3-carboxylic acid^23^, which was the first report regarding the occurrence of alkaloids in *P. asiatica*. As part of our search for biologically active natural products from TCMs, further investigation of the seeds of *P. asiatica* led to the discovery of plasiatine (**1**), an unprecedented heteromeric structure with a C_19_N_2_ skeleton that is composed of an indole analogue and a phenylpropanoid moiety (Fig. 1). Bioactive assay showed that **1** potently promoted the activity of Shp2. Herein, we reported the isolation, structural elucidation, and bioactivity evaluation of compound **1**.

## Results and Discussion

Compound **1**, a white amorphous powder, presented a pseudomolecular ion peak at *m*/*z* 405 [M+Na]^+^ in the positive ESIMS, and its HREIMS data further afforded a molecular formula of C_20_H_18_N_2_O_6_ (*m/z* 382.1160, cacld for C_20_H_18_N_2_O_6_ 382.1165), requiring 13 degrees of unsaturation. The IR spectrum showed characteristic bands at 1720, 1629, 1518, and 1469 cm^−1^ for the carbonyl groups and aromatic rings. In the ^1^H NMR spectrum (Table 1), a methoxyl group (*δ*_H_ 3.78) and two methines (*δ*_H_ 3.45 and one oxygenated at *δ*_H_ 5.44) were observed. A pair of AB doublet appeared at *δ*_H_ 3.03 (d, *J* = 16.6 Hz) and 2.83 (d, *J* = 16.6 Hz), indicating the existence of an isolated methylene. Three typical olefinic proton resonances at *δ*_H_ 6.90 (d, *J* = 1.6 Hz), 6.78 (dd, *J* = 8.1, 1.6 Hz), and 6.72 (d, *J* = 8.1 Hz) could be ascribed to a 1,3,4-trisubstituted benzene ring, and the remaining two olefinic singlets at *δ*_H_ 7.03 and 6.86 might be assigned to one 1,2,4,5-tetrasubstituted or two 1,2,3,4,5-pentasubstituted aromatic rings. The ^13^C NMR and DEPT spectra (Table 1) showed 20 carbon signals, corresponding to one methoxy, two methylenes (including one oxygenated), seven methines (including five olefinic methines and one oxygenated), and 10 quaternary carbons (including eight olefinic, one carbonyl, and one oxygenated). By analysis of the HSQC spectrum, all proton signals were unambiguously assigned to their respective carbons.

**Figure 2:**
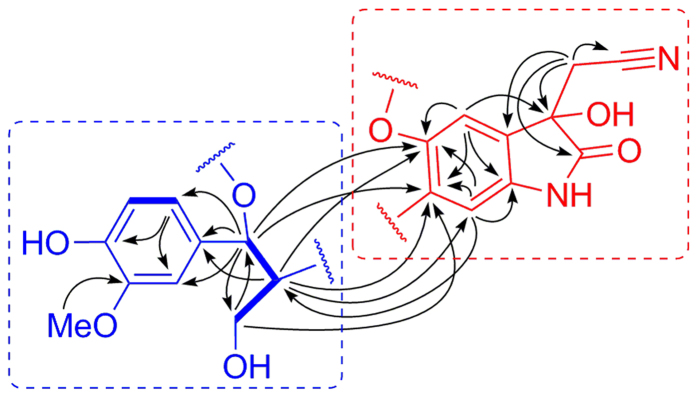
Key ^1^H–^1^H COSY (─) and HMBC (H→C) correlations of 1.

Its 1D NMR data (Table 1) showed that the characteristic signals for an oxoindoline (*δ*_C_ 179.3, C-2; 74.3, C-3; 131.0, C-4; 107.1, C-5; 157.3, C-6; 131.0, C-7; 108.8, C-8; 135.8, C-9) and a cyanomethyl (*δ*_C_ 27.3, C-10; 117.3, C-11) could be distinguished, indicating that **1** containing a 2-(3-hydroxy-2-oxoindolin-3-yl) acetonitrile shared a substructural similarity with plasiaticines B–D^23^. The HMBC correlations (Fig. 2) from H-5 (*δ*_H_ 7.03, s) to C-3 (*δ*_C_ 74.3) and from H_2_-10 (*δ*_H_ 2.83, d, *J* = 16.6 Hz; 3.03, d, *J* = 16.6 Hz) to C-2 (*δ*_C_ 179.3), C-3, and C-4 (*δ*_C_ 131.0) permitted the location of a hydroxyl and a cyanomethyl group at C-3. Correlations from both of H-5 and H-8 (*δ*_H_ 6.86, s) to C-6 (*δ*_C_ 157.3) and C-7 (*δ*_C_ 131.0) showed that C-6 was oxygenated and C-7 was substituted. Thus, the east part of the compound **1** was established to be 2-(3-hydroxy-2-oxoindolin-3-yl)acetonitrile (Fig. 2).

The 1,3,4-trisubstituted benzene ring with a methoxy and a hydroxyl at C-3′ and C-4′, respectively, was supported by the HMBC correlations from OMe (*δ*_H_ 3.78) to C-3′ (*δ*_C_ 149.1) and from H-6′ (*δ*_H_ 6.78) to C-4′ (*δ*_C_ 147.5), together with the ROESY correlation of OMe with H-2′ (*δ*_H_ 6.90, d, *J* = 1.6 Hz). In addition, the ^1^H–^1^H COSY correlations of H-7′/H-8′/H-9′ along with the HMBC correlations from H-6′ to C-1′ (*δ*_C_ 134.6) and from H-7′ (*δ*_H_ 5.44, d, *J* = 6.1 Hz) to C-1′, C-2′ (*δ*_C_ 110.4), C-6′ (*δ*_C_ 119.6), and C-9′ (*δ*_C_ 64.8) demonstrated that the west part of the compound **1** was phenylpropanoid unit (Fig. 2).

The carbon-carbon connection of C-7 and C-8′ was evidenced by the key HMBC correlations from H-8′ (*δ*_H_ 3.45, m) to C-6, C-7, and C-8 (*δ*_C_ 108.8) and from H-8 to C-8′ (*δ*_C_ 55.1) (Fig. 2). Meanwhile, given the deductive formula, the remaining one degree of unsaturation was fulfilled by the establishment of a five-membered oxygen-containing ring flanking the oxoindoline, which was supported by the HMBC correlations from H-7′ to C-6 and C-7 (Fig. 2).

The configuration of C-3 in the oxoindoline could be readily elucidated on the basis of the experimental electronic circular dichroism (ECD) spectrum. Two negative Cotton effects (CEs) at 241 and 289 nm and two positive CEs at 211 and 271 nm in its experimental ECD spectrum, which were in accord with those of plasiaticines B–D^23^ and opposite to that of (+)-(*S*)-2-(3,4-dihydroxy-2-oxoindolin-3-yl)acetonitrile^24^, suggested that C-3 was *R*-configuration. In addition, the ROESY correlation of H-7′ with H_2_-9′ indicated that H-7′ and H-8′ were positioned on the opposite face of the molecule. However, the absolute configurations of C-7′ and C-8′ could not be established because the relative configuration of C-3 relative to those of C-7′ and C-8′ could not be determined by ROESY spectrum. After numerous unsuccessful attempts to obtain a suitable crystal for X-ray diffraction analysis, theoretical calculation studies for ^13^C NMR and ECD spectra of a pair of stereoisomers, (3*R*, 7′*R*, 8′*S*)-**1** and (3*R*, 7′*S*, 8′*R*)-**1**, were perform using Gaussian 09 so as to provide further evidence to confirm the unprecedented skeleton and to determine the absolute configuration.

**Figure 3:**
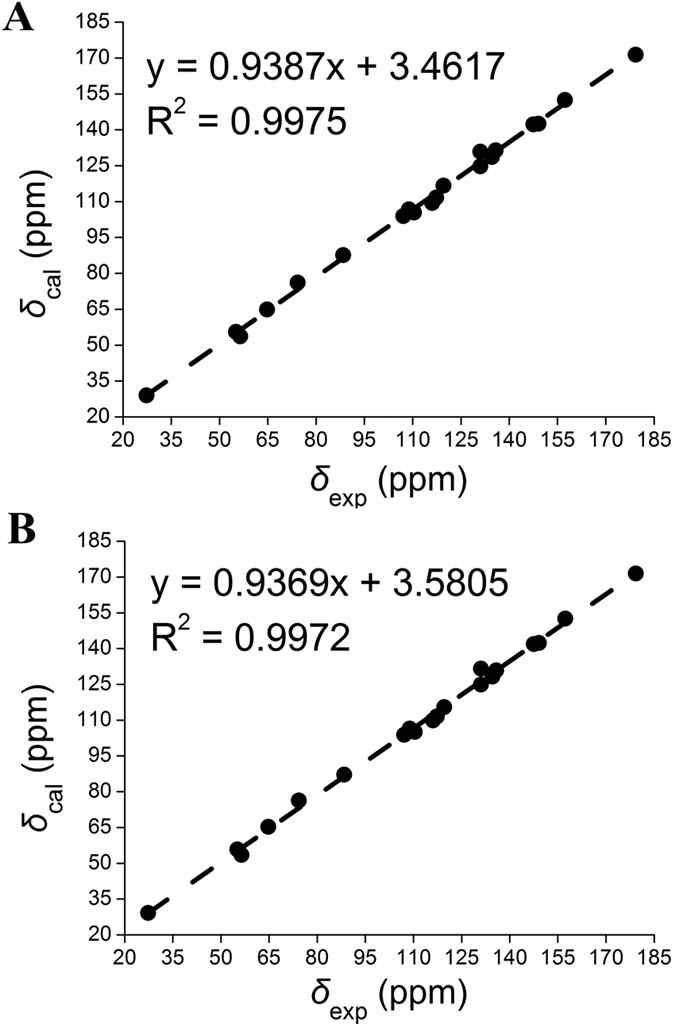
Regression analysis of experimental versus calculated ^13^C NMR chemical shifts of (**A**) (3*R*, 7′*R*, 8′*S*)-**1** and (**B**) (3*R*, 7′*S*, 8′*R*)-**1**, respectively. Linear fitting was shown as a dashed line.

The ^13^C chemical shift predictions of both structures obtained by the gauge-independent atomic orbital method at MPW1PW91-SCRF/6-31 G(d,p) level with the polarizable continuum model (PCM) in MeOH solvent agreed well with the recorded shifts (Table 1 and Fig. 3), and the mean absolute error, the corrected mean absolute error, and *R*^2^ (Fig. 3) were 3.31 ppm, 1.46 ppm, and 0.9975 for (3*R*, 7′*R*, 8′*S*)-**1**, and 3.39 ppm, 1.44 ppm, and 0.9972 for (3*R*, 7′*S*, 8′*R*)-**1**, respectively. Thus, the ^13^C NMR calculation evidenced the proposed skeleton determined by our NMR analysis.

**Figure 4:**
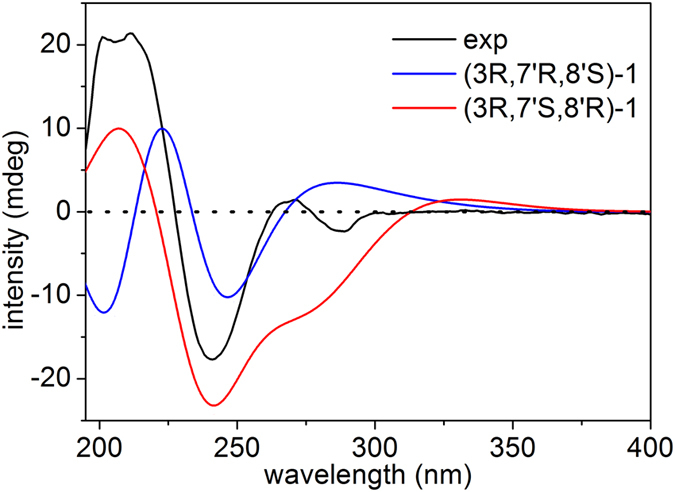
Experimental ECD spectrum of 1 (black) and calculated ECD spectra of (3*R*, 7′*S*, 8′*R*)-1 (red) and (3*R*, 7′*R*, 8′*S*)-1 (blue) in MeOH.

Subsequently, the absolute configurations of the stereogenic centers (C-7′ and C-8′) in the western hemisphere was elucidated by ECD calculation using time-dependent density-functional theory at B3LYP-SCRF/6-31 + G(d,p) level with PCM in MeOH solvent. The calculated ECD curves of two stereoisomers (3*R*, 7′*R*, 8′*S*)-**1** and (3*R*, 7′*S*, 8′*R*)-**1** were compared with the experimental spectrum. The wave troughs of the calculated curves of (3*R*, 7′*R*, 8′*S*)-**1** and (3*R*, 7′*S*, 8′*R*)-**1** around 240 nm were close to the experimental ECD band at 241 nm (Fig. 4). However, (3*R*, 7′*R*, 8′*S*)-**1** had a wave trough around 205 nm, while (3*R*, 7′*S*, 8′*R*)-**1** exhibited a wave crest at the same position which corresponded well with the experimental curve. Molecular orbital (MO) analysis (Figure S5) suggested that the negative CE at 241 nm in the experimental spectrum could be ascribed to the negative rotatory strength at 237.4 nm, which was caused by the electronic transitions from MO96 to MO101 in the 2-oxoindoline moiety; and the positive CE around 205 nm could be assigned to two positive rotatory strengths at 206.7 and 214.9 nm, which correlated with the electronic transitions from MO100 to MO114 and from MO99 to MO108, respectively. Therefore, the absolute configuration of compound **1** was established as 3*R*, 7′*S*, and 8′*R*.

**Figure 5:**
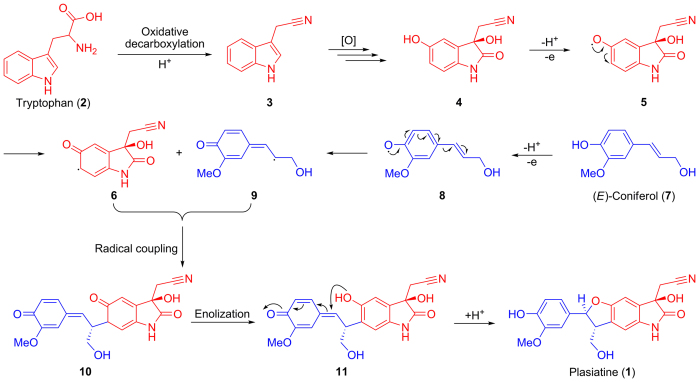
Hypothetical biogenetic pathway to plasiatine (1).

Indole alkaloids, which may occupy a quarter of alkaloids from natural resources, represent a particular and vast group of natural products^25^. Generally, they are present as prenyl hybridsthat are biosynthetically derived from tryptophan or its precursors fused with isopentenyl pyrophosphate^26^, dimethylallyl pyrophosphate^26^, geranyl pyrophosphate^27^, or farnesyl pyrophosphate^28^,^29^, while other forms of indole hybrids are exceedingly rare. From a literature research, only one reference reports that ipobscurines B–D feature an indole analogue conjugated with two phenylpropanoid moieties via C–O and C–N bonds^9^; thus plasiatine (**1**) is the first heteromeric construction formed by a crucial carbon–carbon connection between an indole and a phenylpropanoid moiety, and its biogenetic pathway was also proposed (Fig. 5).

**Figure 6:**
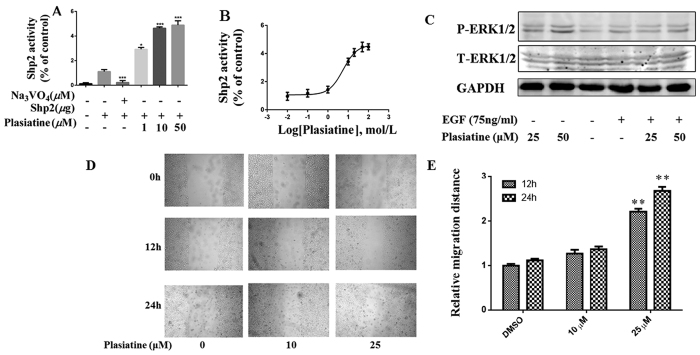
(**A**) The activity of Shp2 was determined after GST-Shp2 recombinant protein (0.1 *μ*M) treated with Na_3_VO_4_ (50 *μ*M) and different concentrations of plasiatine (**1**), respectively. (**B**) Presents the concentration-response curve of plasiatine (**1**) for activation of Shp2 activity using GST-Shp2 recombinant protein. (**C**) HEK293A cells were treated with 25 *μ*M or 50 *μ*M plasiatine (**1**) for 2 h with or without 75 ng/mL EGF pretreatment. The cell lysates were immunoblotted with an anti-pErk1/2 (P-ERK1/2), total Erk1/2 (T-ERK1/2) or anti-GAPDH antibody, respectively. (**D**,**E**) Effect of plasiatine (**1**) on hepatocelluar HepG2 cells migration. Panel E showed quantification analysis of wound healing assay from triplicate measurements ratio of the migration distance in each group divided by the migration distance in control (DMSO) cells. Bars represented the mean values ± SD of three experiments. Scale bar, 250 μm. ^∗^*p* < 0.05, ^∗∗^*p* < 0.01, and ^∗∗∗^*p* < 0.001 vs control.

**Figure 7:**
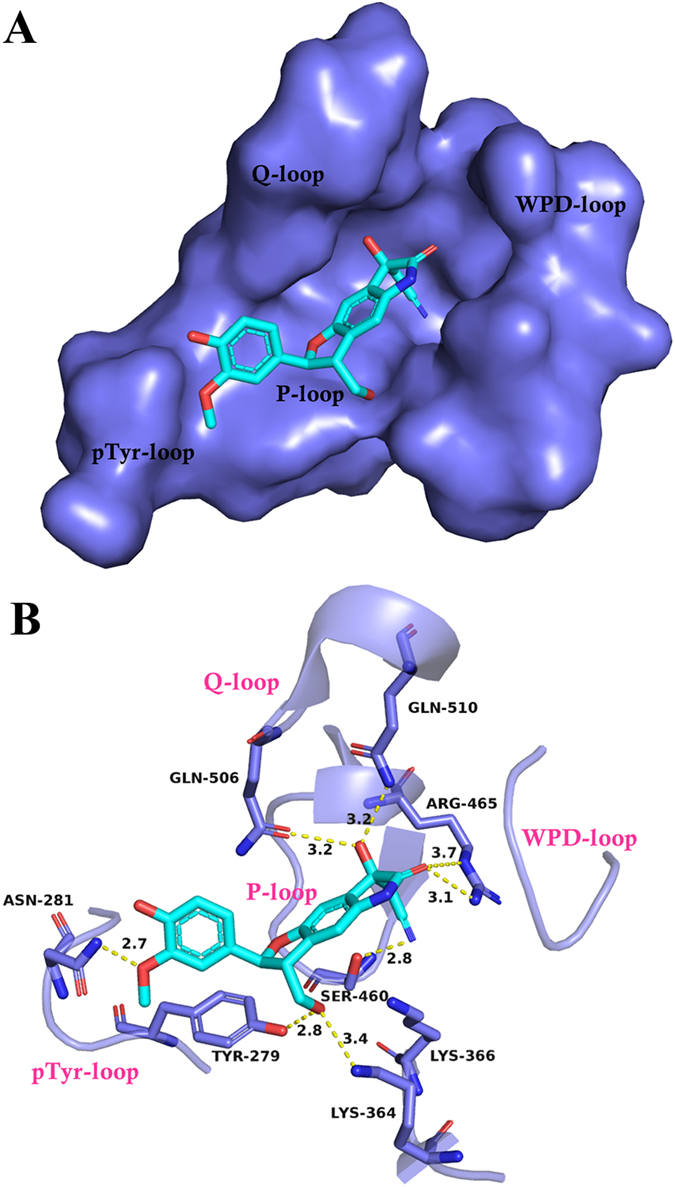
The predicted binding mode of plasiatine (1) in the catalytic domain of Shp2 (PDB entry: 4PVG). (**A**) The binding mode revealed by molecular docking. (**B**) Interaction details of plasiatine (**1**) and Shp2. Plasiatine (**1**) (cyan carbon) and residues in contact with 1 (blue carbon) were presented in sticks. H-bond interactions were highlighted in yellow dashed lines.

Our present study revealed that plasiatine (**1**) potently promoted the phosphatase activity of Shp2 in a concentration-dependent manner, with an EC_50_ value of 0.97 *μ*M (Na_3_VO_4_ as a negative control, Fig. 6A,B). Further study found that plasiatine (**1**) enhanced the phosphorytion of Erk1/2 in the present or absent of EGF, a known downstream target of Shp2^6^ (Fig. 6C). The wound-healing assay showed that plasiatine (**1**) at a concentration of 25 *μ*M significantly promoted hepatocellular HepG2 cells migration (Fig. 6D,E). To map the binding site of plasiatine (**1**), we built complex structure of the compound with Shp2 by using computational docking method. As shown in Fig. 7, plasiatine (**1**) fitted well in the active site and the complex was stabilized by extensive hydrogen bonding and hydrophobic interaction. The model revealed that the oxoindoline moiety penetrated into the active site PTP signature motif (VHCSAGIGRTG)^30^. The oxoindoline carbonyl, acetonitrile, and hydroxyl group exhibited hydrogen bonding interactions with atoms of P-loop residues (Arg465 and Ser460) and Q-loop residues (Gln506 and Gln510). The dihydrofuran and hydroxymethyl group were located at the entrance of the active site and interacted with residues Lys364 and Tyr279. The methoxyl group formed hydrogen bonds with the side-chain NH_2_ group of Asn281 that is non-conserved PTP residues located adjacent to the phosphotyrosine recognition loop. The interaction between aromatic rings of the compound and the protein contributed to the binding through hydrophobic stabilization. The computational docking result was in good agreement with the bioactivity evaluation and obviously disclosed the detailed interactions between plasiatine (**1**) and Shp2. Compounds with oxindole scaffolds were identified to selectively inhibit Shp2 activity^31^. Plasiatine (**1**) and the previously reported oxindole compounds penetrate into the active site PTP signature motif through hydrogen bonding interactions with atoms of PTP loop residues (Arg465 and Ser460). In contrast to the oxindole compounds, plasiatine (**1**) also interacts with Gln506 and Tyr279, and mutations of these two residues are found to participate in the incidence of Noonan syndrome and juvenile myelomonocytic leukemia by increasing Shp2 activity^32^. Therefore, it is possible that plasiatine (**1**) promotes Shp2 activity through interaction with these two residues. Plasiatine (**1**) also forms hydrogen bonds with Asn282 adjacent to the phosphotyrosine recognition loop. It will be of interest to further investigate whether plasiatine (**1**) increases the combination with the substrate tyrosine to promote the Shp2 activity.

To our knowledge, this study represents the first identification of a small molecule Shp2 activator and opens a new way in Shp2-associated oncogenic mechanism research. This compound warrants further investigation as a novel pharmaceutical tool to study the function of Shp2 in tumorigenesis and facilitate the rational design of Shp2 activating agents.

## Methods

### General Experimental Procedures

Optical rotations were measured on a JASCO P-1020 digital polarimeter. UV data were obtained on a Shimadzu UV2401PC spectrophotometer. Experimental ECD spectra were measured on a Chirascan instrument. A Bruker Tensor-27 spectrophotometer was used for scanning IR spectroscopy with KBr pellets. 1D and 2D NMR spectra were recorded on Bruker DRX-500 spectrometer. Unless otherwise specified, chemical shifts (*δ*) were expressed in ppm with reference to the solvent signals. ESIMS were performed on Waters Xevo TQ-S. HREIMS were performed on Waters AutoSpec Premier P776. Column chromatography was performed with silica gel (200–300 mesh; Qingdao Marine Chemical, Inc., Qingdao, P. R. of China), MCI gel (75–150 *μ*m, Mitsubishi Chemical Corporation, Tokyo, Japan), and Sephadex LH-20 gel (40–70 *μ*m, Amersham Pharmacia Biotech AB, Uppsala, Sweden). Semipreparative HPLC was performed on an Agilent 1200 liquid chromatograph with a Zorbax SB-C_18_, 9.4 mm × 25 cm column. Fractions were monitored by TLC and spots were visualized by heating silica gel plates sprayed with 10% H_2_SO_4_ in EtOH. All solvents including petroleum ether (60–90 °C) were distilled prior to use.

### Plant Material

The seeds of *P. asiatica* were purchased from Juhuacun Traditional Chinese Medicine Market, Kunming, Yunnan Province, People’s Republic of China, in August 2011. A voucher specimen (No. KIB 2011-08-11) was identified by Mr. Yu Chen and was deposited at the State Key Laboratory of Phytochemistry and Plant Resources in West China, Kunming Institute of Botany, Chinese Academy of Sciences.

### Extraction, Isolation, and Purification

The air-dried powders seeds of *P. asiatica* (10 kg) were extracted with 70% aqueous acetone (3 × 30 L) at room temperature and concentrated in vacuo to give a crude extract which was partitioned between H_2_O and EtOAc. The EtOAc extract (285 g) was chromatographed on a silica gel column eluted with gradient CHCl_3_–Me_2_CO (1:0 to 1:0) to afford fractions A–E. Fraction D (9.3 g) was chromatographed on MCI gel CHP 20 P eluted with gradient MeOH–H_2_O (1:4 to 1:0) to yield seven fractions, D1–7. Subfraction D3 (316 mg) was purified by Sephadex LH-20 (CHCl_3_–MeOH, 1:1) and semipreparative HPLC (MeCN–H_2_O, 15:85) to afford compound **1** (7 mg).

### ^13^C NMR and ECD Calculations and Molecular Orbital Analysis

The theoretical calculations of compound **1** were carried out using Gaussian 09^33^. Conformational analysis was initially performed using Maestro 9.0 with the OPLS_2005 force field. The conformers were optimized at B3LYP/6-31 G(d) level. Room-temperature equilibrium populations were calculated according to Boltzmann distribution law. The optimized conformation geometries, thermodynamic parameters, and populations of all conformations were provided in Figures S11 and S12 and Tables S1, S2, S5 and S6 in the Supplementary Information.

^13^C NMR shielding constants of compound **1** were calculated with the GIAO method^34^ at MPW1PW91-SCRF/6-31 G(d,p) level with the polarizable continuum model (PCM) in MeOH solvent. The shielding constants so obtained were converted into chemical shifts by referencing to TMS at 0 ppm (*δ*_cal_ = *σ*_TMS_ − *σ*_cal_), where the *σ*_TMS_ was the shielding constant of TMS calculated at the same level. For each stereoisomer, the parameters *a* and *b* of the linear regression *δ*_cal_ = *aδ*_exp_ + *b*; the correlation coefficient, *R*^2^; the mean absolute error (MAE) defined as Σ_n_ |*δ*_cal_ − *δ*_exp_|/*n*; the corrected mean absolute error (CMAE), defined as Σ_n_ |*δ*_corr_ − *δ*_exp_|/*n*, where *δ*_corr_ = (*δ*_cal_ − *b*)/*a* and therefore corrects for systematic errors were presented.

The theoretical calculations of ECD were performed using TDDFT^35^,^36^ at B3LYP/6-31 + G(d,p) level in MeOH with PCM. The ECD spectra of compound **1** were obtained by weighing the Boltzmann distribution rate of each geometric conformation. The ECD spectra are simulated by overlapping Gaussian functions for each transition according to


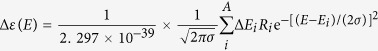


where *σ* represents the width of the band at 1/*e* height, and Δ*E*_*i*_ and *R*_*i*_ are the excitation energies and rotational strengths for transition *i*, respectively. *σ* = 0.20 eV and *R*^velocity^ were used in this work.

The orbital information (NBO plot files) was generated by NBO program of Gaussian 09^37^. The predominantly populated conformers were selected for molecular orbital (MO) analysis. NBO plot files were used to generate corresponding Gaussian-type grid file by Multiwfn 2.4^38^. After that, the isosurface of generated grid date was afforded by VMD software^39^.

### Shp2 Activity Assay

Plasmids for expression of glutathione *S*-transferase (GST)-PTP fusion proteins of human Shp2 (residues 205–597) were constructed in pGEX-4T1 by PCR subcloning techniques. All constructs were verified by DNA sequencing. GST- Shp2 fusion proteins were expressed in *Escherichia coli* BL21 and affinity purified with glutathione Sepharose. After elution from glutathione affinity columns, GST-fusion proteins were dialyzed with dialysis buffer (12.5 mM Tris-Cl, pH 7.5, 25 mM NaCl, 1 mM dithiothreitol, and 0.1% β-mercaptoethanol) at 4 °C over night and then stored in dialysis buffer plus 20% glycerol at −80 °C.

Shp2 activity was measured as described previously^6^. Using the 8-difluoro-4-methylumbelliferyl phosphate (DiFMUP; Invitrogen, Carlsbad, CA) as the substrate, reaction buffer contained 50 mM Hepes (pH 7.0), 150 mM NaCl, 0.05% Tween 20, 2 mM dithiothreitol, 1 mM EDTA, 20 μM DiFMUP, 0.1 μM GST-Shp2, and different concentrations of test compound or dimethyl sulfoxide (solvent) in a total reaction volume of 100 *μ*L in black 96-well plates. Reaction was initiated by addition of DiFMUP, and the incubation time was 30 min at 37 °C. DiFMUP fluorescence signal was measured at an excitation of 355 nm and an emission of 455 nm with a plate reader (Thermo Scientific Verioskan Flash).

### Western Blotting

Cells cultured in 60-mm dishes were lysed with RIPA Lysis Buffer (Beyotime, Haimen, China) supplemented with protease inhibitor cocktail (Sigma). Equal protein content of total cell lysates were mixed in loading buffer, boiled for 5 min, and then subjected to SDS-PAGE gel (10%) electrophoresis along with EasySee western marker (TransGen Biotech, Beijing, China). After electrophoresis, proteins were blotted onto nitrocellulose membranes and blocked with 5% bovine serum albumin, probed with a primary antibody at 4 °C overnight, followed by a horseradish peroxidase-conjugated secondary antibody (Santa Cruz Biotechnology, Santa Cruz, CA, USA), then enhanced chemiluminesence detection (Amersham Bioscience, Piscataway, NJ).

### *In vitro* Wound-healing Assay

Wound healing experiments were performed as described previously^40^. In brief, 1 × 10^5^ HepG2 cells per well were plated in 6-well plates.The confluent HepG2 cells were scratched with a 200 *μ*L disposable plastic pipette tip and were allowed to migrate towards the wound. To examine migration in HepG2 cells, the medium was replaced with that supplemented with 2% FBS either in the presence or absence of plasiatine (**1**), followed by scratching. Wound closure rates were expressed as a percentage of the migratory distance in control cells (100%).

### Preparation of Protein and Molecular Docking

The 3D crystal structure of Shp2 was retrieved from the Protein Data Bank (PDB entry: 4PVG). The program Maestro 9.0 (Schrödinger, Inc.) was used for this assessment. All water molecules were removed from the structure of the complex. Hydrogen atoms and charges were added during a brief relaxation that was performed using the “Protein Preparation Wizard” workflow. After optimizing the hydrogen bond network, the crystal structure was minimized using the OPLS_2005 force field with the maximum root mean square deviation (RMSD) value of 0.3 Å. The grid-enclosing box was centered on the ligand L88N79 in the refined crystal structure as described above. This domain has been identified as the PTP catalytic domain. The three-dimensional structure of plasiatine (**1**) was generated with the Ligprep module. Docking process was performed using Glide with default docking parameter settings with standard precision (SP) mode.

Plasiatine (**1**) White amorphous powder; 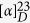 −37.1 (*c* 0.12, MeOH); UV (MeOH) *λ*_max_ (log *ε*) 203 (3.91), 277 (3.33), 320 (2.63) nm; ECD (*c* 0.09 MeOH) *λ*_max_ (Δ*ε*) 211 (+21.36), 241 (−17.70), 271 (+1.44), 289 (−2.34) nm; IR (KBr) *ν*_max_ 3429, 1720, 1629, 1469, 1274 cm^−1^; ^1^H and ^13^C NMR data, see Table 1; positive ESIMS *m*/*z* 405 (100) [M+Na]^+^; HREIMS *m*/*z* 382.1160 [M]^+^ (calcd for C_20_H_18_N_2_O_6_, 382.1165).

## Additional Information

**How to cite this article**: Gao, Z.-H. *et al*. Plasiatine, an Unprecedented Indole–Phenylpropanoid Hybrid from *Plantago asiatica* as a Potent Activator of the Nonreceptor Protein Tyrosine Phosphatase Shp2. *Sci. Rep.* **6**, 24945; doi: 10.1038/srep24945 (2016).

## Supplementary Material

Supplementary Information

## Figures and Tables

**Figure 1:**
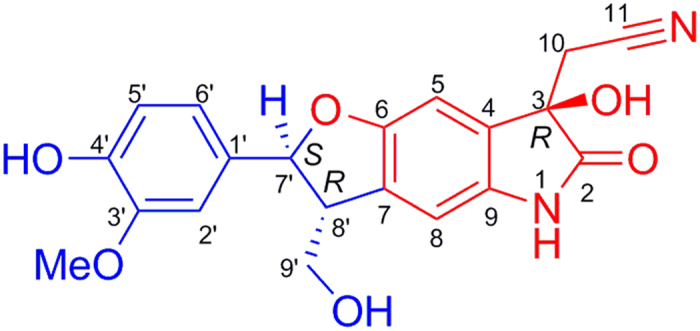
Structure of plasiatine (1).

**Table 1:** 1H and ^13^C NMR data for plasiatine (**1**) (*δ* in ppm, *J* in Hz) in methanol-*d*_4_.

No.	1		(3*R*, 7′*R*, 8′*S*)-1^c^	(3*R*, 7′*S*, 8′*R*)-1^c^
	*δ*_H_^a^	*δ*_C_^b^		
2	–	179.3, C	171.3	171.5
3	–	74.3, C	76.1	76.3
4	–	131.0, C	124.7	124.9
5	7.03 (s)	107.1, CH	103.9	103.8
6	–	157.3, C	152.5	152.5
7	–	131.0, C	130.8	131.6
8	6.86 (s)	108.8, CH	106.8	106.5
9	–	135.8, C	131.4	130.8
10	3.03 (d, 16.6)	27.3, CH_2_	29.0	29.2
	2.83 (d, 16.6)	–	–	–
11	–	117.3, C	111.7	111.6
1′	–	134.6, C	128.6	128.3
2′	6.90 (d, 1.6)	110.4, CH	105.4	105.0
3′	–	149.1, C	142.6	142.4
4′	–	147.5, C	142.3	141.9
5′	6.72 (d, 8.1)	116.1, CH	109.4	109.9
6′	6.78 (dd, 8.1, 1.6)	119.6, CH	116.6	115.4
7′	5.44 (d, 6.1)	88.4, CH	87.6	87.1
8′	3.45 (m)	55.1, CH	55.5	55.8
9′	3.77 (m)	64.8, CH_2_	64.9	65.3
OMe-3′	3.78 (s)	56.4, CH_3_	53.7	53.5

^a^Recorded in 500 MHz.

^b^Recorded in 125 MHz.

^c^Calculated ^13^C NMR chemical shift.
