# Supplementary material for: Plasiatine, an Unprecedented Indole–Phenylpropanoid Hybrid from Plantago asiatica as a Potent Activator of the Nonreceptor Protein Tyrosine Phosphatase Shp2
Source: Sci Rep. 2016 Apr 22;6:24945. doi: 10.1038/srep24945 (PMC4840323; doi:10.1038/srep24945)
Supplement: Supplementary Information [file srep24945-s1.pdf]

Supplementary Information for

Plasiaticine E, an Unprecedented Indole–Phenylpropanoid Hybrid from *Plantago asiatica* as a Potent Activator of the Nonreceptor Protein Tyrosine Phosphatase Shp2

Zhong-Hua Gao[1,+], Yi-Ming Shi[2,+], Zhe Qiang[3], Xia Wang[4], Shan-Zhai Shang[1], Yan Yang[1], Bao-Wen Du[3], Hui-Pan Peng[3], Xu Ji[1], Honglin Li[4,*], Fei Wang[3,*], and Wei-Lie Xiao[1,*]

[1]State Key Laboratory of Phytochemistry and Plant Resources in West China, Kunming Institute of Botany, Chinese Academy of Sciences, Kunming, 650201, P. R. China
[2]Guangdong Provincial Academy of Chinese Medical Sciences, Guangzhou, 510006, P. R. China
[3]Chengdu Institute of Biology, Chinese Academy of Sciences, Chengdu, 610041, P. R. China
[4]Shanghai Key Laboratory of New Drug Design, School of Pharmacy, East China University of Science and Technology, Shanghai, 200237, P. R. China
*xwl@mail.kib.ac.cn
*wangfei@cib.ac.cn
*hlli@ecust.edu.cn
[+]these authors contributed equally to this work

Content

Computational Data of Plasiatine (1) **1**
Molecular Orbital (MO) Analysis of Plasiatine (1) **13**
NMR, MS, IR, UV, and ECD Spectra of Plasiatine (1) **14**

Computational Data of Plasiatine (1)

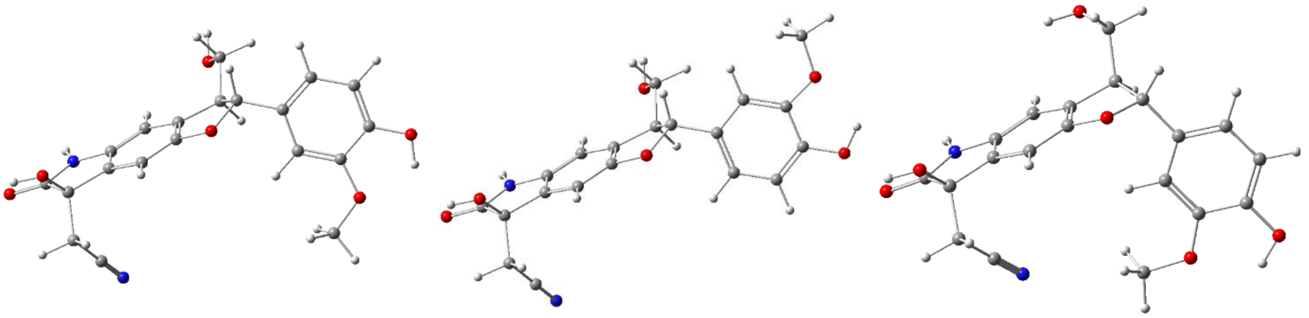

a **b** **c**

Figure S1. Optimized geometries of conformers **a-c** of (3*R*,7'*R*,8'*S*)-**1** at the B3LYP/6-31G(d) level in the gas phase.

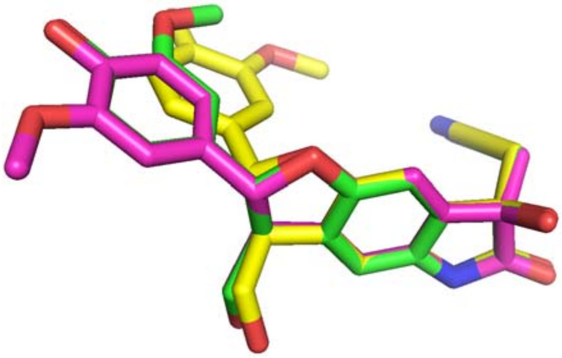

Figure S2. Overlay of conformers **a**–**c**.

Table S1. Important thermodynamic parameters (a.u.) of the optimized (3*R*,7'*R*,8'*S*)-**1** (conforrmers **a-c**) at B3LYP/6-31G(d) level in the gas phase.[a]

species	*E*	*E'*=*E*+*ZPE*	*H*	*G*
**a**	-1333.388231	-1333.413421	-1333.387287	-1333.470363
**b**	-1333.386427	-1333.411644	-1333.385483	-1333.468800
**c**	-1333.388988	-1333.413911	-1333.388044	-1333.469473

[a]*E* = total energy; *E'* = total energy with zero point energy (*ZPE*); *H* = enthalpy; *G* = Gibbs free energy.

Table S2. Conformational analysis of conforrmers **a-c** of (3*R*,7'*R*,8'*S*)-**1**.

species	ΔE[a]	$P_E\%$[b]	$\Delta E'$[c]	$P_{E'}\%$[d]	ΔG[e]	$P_G\%$[f]
**a**	0.48	29.6	0.31	35.3	0.00	63.3
**b**	1.61	4.4	1.42	5.4	0.98	12.1
**c**	0.00	66.0	0.00	59.3	0.56	24.6

[a]Relative energy, [c]relative energy with *ZPE*, and [e]relative Gibbs free energy in kcal/mol. [b d f] Conformational distribution calculated by using the respective parameters above at B3LYP/6-31G(d) level in the gas phase. (T=298 K)

Table S3. Key transitions, oscillator strengths, and rotatory strengths in the ECD spectra of conformers **a**–**c** at B3LYP-SCRF/6-31+G(d,p)//B3LYP/6-31G(d) level with PCM in MeOH.

species	exited state	*ΔE (eV)*[a]	*λ (nm)*[b]	*f*[c]	R_{vel}[d]	R_{len}[e]
	100->101	3.9354	315.05	0.0330	4.6273	5.1984
	99->101	4.3665	283.94	0.0018	0.8808	0.8594
	100->102	4.5276	273.84	0.2421	-1.8858	-2.4431
	100->103	4.7656	260.16	0.0232	12.4793	11.2982
	99->103	4.8576	255.24	0.0645	-0.5481	-0.6651
	99->102	4.9300	251.49	0.0045	-6.6380	-6.8595
	100->104	5.0292	246.53	0.0001	-0.0435	-0.1081
	98->101	5.1349	241.46	0.0101	-16.4942	-17.4659
	100->105	5.1735	239.65	0.0312	-2.3871	-2.4938
	96->101	5.2215	237.45	0.0081	-31.3189	-31.7071
	100->106	5.2494	236.19	0.0018	1.2873	2.4842
	99->106	5.2698	235.27	0.0004	1.4859	1.3894
	100->108	5.3742	230.70	0.0203	2.9374	2.8672
	99->105	5.4479	227.58	0.2202	18.9508	19.2769
	100->109	5.4797	226.26	0.0018	0.6456	0.6939
**a**	100->110	5.4997	225.44	0.0576	-20.8651	-20.5622
	99->104	5.5262	224.36	0.0012	5.6460	5.5241
	97->101	5.5769	222.32	0.3565	58.1414	61.5496
	100->110	5.6441	219.67	0.0194	-1.3384	-0.8390
	100->111	5.6764	218.42	0.0202	-7.3644	-7.9405
	100->111	5.6897	217.91	0.0233	17.5350	16.8108
	98->102	5.7014	217.46	0.0052	-7.0869	-6.7304
	100->112	5.7604	215.24	0.0116	-9.2796	-8.8821
	99->109	5.7929	214.03	0.0061	5.4266	5.8954
	99->108	5.8101	213.39	0.0021	-0.6082	-0.6823
	99->107	5.8464	212.07	0.0058	6.1006	6.5699
	100->113	5.8906	210.48	0.0594	-2.6192	-2.3789
	95->101	5.9632	207.91	0.0004	-1.5522	-0.9603
	99->111	5.9741	207.54	0.0205	-12.6657	-14.8123
	100->114	6.0210	205.92	0.0111	-2.3240	-1.9953
	100->101	3.9331	315.23	0.0337	4.5958	4.7762
	99->101	4.3514	284.93	0.0006	0.0738	-0.0185
	100->102	4.5265	273.91	0.2613	-13.4568	-13.9184
	100->103	4.8430	256.01	0.0437	1.4668	1.7822
	99->102	4.9012	252.96	0.0056	6.8060	6.3435
**b**	100->104	4.9213	251.94	0.0110	6.7063	6.4088
	99->103	5.0183	247.06	0.0108	-0.4151	-0.4359
	100->105	5.0349	246.25	0.0001	0.0468	-0.0343
	98->101	5.2024	238.32	0.0009	6.7854	6.6901
	100->106	5.2180	237.61	0.0028	1.6592	0.6232
	96->101	5.2247	237.30	0.0173	-61.7838	-62.0064

	100->107	5.2659	235.45	0.0031	-9.0525	-9.3839
	99->104	5.3395	232.20	0.1600	8.9693	9.6347
	100->108	5.3731	230.75	0.0212	12.6905	13.6136
	100->109	5.4696	226.68	0.0019	-4.9678	-4.3110
	100->110	5.4983	225.49	0.0010	-1.3436	-1.9412
	99->105	5.5343	224.03	0.0309	-2.2231	-2.2524
	97->101	5.5838	222.04	0.4937	-6.4084	-5.4022
	99->106	5.6204	220.60	0.0429	4.3317	4.9378
	100->108	5.6561	219.21	0.0138	14.0134	13.8284
	100->111	5.7064	217.27	0.0182	10.7401	11.1117
	100->112	5.7439	215.85	0.0020	-3.4220	-3.2197
	98->102	5.7642	215.09	0.0059	6.5886	6.8870
	100->112	5.7845	214.34	0.0025	4.1777	4.0893
	99->107	5.8182	213.10	0.0032	-0.5427	-0.5918
	99->110	5.8375	212.39	0.0120	-3.5620	-3.4785
	100->113	5.8848	210.68	0.0457	2.8419	2.1400
	99->111	5.9588	208.07	0.0081	-5.0886	-5.2996
	95->101	5.9636	207.90	0.0014	0.7780	1.4276
	100->114	5.9792	207.36	0.0486	2.3954	2.4198
**c**	100->101	3.8908	318.66	0.0339	-4.2338	-3.2277
	99->101	4.2154	294.12	0.0161	5.4411	5.0068
	100->102	4.4752	277.04	0.2515	43.8082	45.0827
	99->102	4.7436	261.37	0.0051	-10.7063	-11.2066
	100->103	4.7534	260.84	0.0092	-21.6714	-21.5717
	99->103	4.8488	255.70	0.0518	18.1281	18.0488
	98->101	4.9838	248.77	0.0059	1.8146	1.0260
	100->104	5.0364	246.18	0.0004	-1.3346	-1.4125
	100->105	5.1766	239.51	0.0353	-24.3758	-24.7715
	96->101	5.2054	238.18	0.0081	-36.3074	-37.2570
	100->106	5.2298	237.07	0.0017	-2.2078	-1.1601
	99->107	5.2492	236.20	0.0006	1.6654	1.5820
	100->108	5.3993	229.63	0.0151	2.7916	2.6977
	99->105	5.4231	228.62	0.1989	61.1221	61.8027
	99->104	5.4779	226.33	0.0266	-24.2704	-22.8633
	98->102	5.4901	225.83	0.0663	34.8336	37.0470
	98->102	5.5230	224.49	0.0090	-9.5606	-10.2189
	100->109	5.5380	223.88	0.0080	-0.3165	-0.6607
	97->101	5.5678	222.68	0.1985	6.9756	8.3003
	99->106	5.5898	221.80	0.0251	-15.2249	-15.3247
	100->110	5.6627	218.95	0.0343	1.9707	2.1397
	99->108	5.7334	216.25	0.0009	2.0705	1.6463
	99->110	5.7435	215.87	0.0029	3.1994	3.9047
	100->111	5.8135	213.27	0.0015	-6.3096	-5.8211
	100->112	5.8776	210.94	0.0191	24.1691	24.7840
	99->109	5.8872	210.60	0.0077	-4.2798	-4.2871

	95->101	5.9263	209.21	0.0218	-7.4817	-8.4780
	100->113	5.9754	207.49	0.0593	7.3295	9.3681
	98->103	6.0357	205.42	0.0758	-21.2735	-26.0497
	100->114	6.0524	204.85	0.0700	-100.5624	-103.8279

Table S4. Optimized Z-matrixes of comformers a-c of (3*R*,7'*R*,8'*S*)-**1** in the gas phase (Å) at B3LYP/6-31G(d) level.

a				b			
C	5.362510	0.809285	0.858549	C	3.975902	-1.973071	-1.052000
C	5.528883	-0.420716	0.236078	C	5.192625	-1.467206	-0.603748
C	4.391982	-1.160524	-0.160529	C	5.206015	-0.349588	0.253742
C	3.116209	-0.659681	0.058177	C	4.011051	0.244531	0.648928
C	2.949461	0.588320	0.685573	C	2.784927	-0.259171	0.185993
C	4.074748	1.306515	1.086835	C	2.779907	-1.370046	-0.661433
C	1.568641	1.158749	0.876739	C	1.501138	0.425400	0.579275
C	0.867534	1.676962	-0.420822	C	0.781954	1.223513	-0.555179
O	0.676769	0.150205	1.436045	O	0.531022	-0.551439	1.053856
C	-1.724795	1.487060	-0.915867	C	-1.844347	1.460603	-0.788534
C	-2.877117	0.837791	-0.482874	C	-3.037354	0.907283	-0.333033
C	-2.876122	-0.057495	0.598106	C	-3.073629	-0.150167	0.589214
C	-1.708949	-0.334206	1.302443	C	-1.902836	-0.694356	1.106770
N	-4.188739	0.944249	-1.004617	N	-4.359788	1.279854	-0.674000
C	-5.080984	0.188005	-0.299890	C	-5.288099	0.547285	0.009056
C	-4.274709	-0.601608	0.769370	C	-4.517167	-0.518512	0.837485
C	-4.400548	-2.131423	0.518574	C	-4.903949	-1.949059	0.364877
O	-6.294578	0.138894	-0.412345	O	-6.499302	0.688766	0.038262
O	-4.814694	-0.316575	2.049172	O	-4.881773	-0.387981	2.201652
C	-0.554964	1.212552	-0.206762	C	-0.670206	0.919005	-0.264515
C	-0.557120	0.322467	0.870137	C	-0.709339	-0.131074	0.656437
C	1.058984	3.177838	-0.616089	C	1.169143	2.699059	-0.553566
O	6.775852	-0.919033	0.014801	O	6.361197	-2.051956	-0.985216
O	0.497342	3.528394	-1.876966	O	0.561394	3.311793	-1.685997
C	-3.814760	-2.593064	-0.741448	C	-4.542186	-2.250684	-1.021501
N	-3.351036	-2.951458	-1.743286	N	-4.259224	-2.478638	-2.123787
O	4.700065	-2.361934	-0.749601	O	6.469633	0.043070	0.628982
C	3.630431	-3.188307	-1.190059	C	6.596807	1.141907	1.517667
H	6.244654	1.360759	1.167771	H	3.983454	-2.840604	-1.704246
H	2.240211	-1.230314	-0.228609	H	4.019240	1.095155	1.323193
H	3.954858	2.263215	1.589144	H	1.835361	-1.779802	-1.005706
H	1.614068	1.978693	1.608545	H	1.695251	1.107536	1.421240
H	1.296730	1.179162	-1.301388	H	1.063844	0.813874	-1.534930
H	-1.717816	2.177037	-1.752615	H	-1.810025	2.274291	-1.504713
H	-1.686705	-1.012413	2.149044	H	-1.906550	-1.502712	1.830437
H	-4.472763	1.581820	-1.735941	H	-4.608998	2.065015	-1.260004
H	-3.920791	-2.649657	1.356194	H	-4.420697	-2.668377	1.035126
H	-5.466968	-2.388586	0.529833	H	-5.989456	-2.058573	0.479012
H	-5.781895	-0.274392	1.927291	H	-5.837641	-0.192079	2.204228
H	0.569156	3.722595	0.206766	H	0.832313	3.167754	0.385026
H	2.135653	3.410826	-0.584021	H	2.267259	2.783862	-0.599965
H	6.661991	-1.783481	-0.418432	H	7.080703	-1.553398	-0.559601
H	0.600247	4.484615	-1.996295	H	0.781627	4.255437	-1.672162
H	2.986098	-3.482596	-0.352395	H	6.178869	2.058029	1.080420
H	4.093622	-4.076233	-1.623271	H	7.667206	1.275567	1.683264

H	3.026138	-2.681306	-1.952583	H	6.101804	0.938585	2.475963

**c**			
C	5.163972	0.109651	0.378578
C	4.784919	-1.147418	-0.072855
C	3.412034	-1.452274	-0.215683
C	2.447375	-0.500962	0.085777
C	2.832030	0.775350	0.540889
C	4.187760	1.063332	0.688698
C	1.793173	1.832184	0.835349
C	1.008507	2.348813	-0.413866
O	0.768240	1.304863	1.745969
C	-1.348142	1.427226	-1.180470
C	-2.439202	0.688217	-0.727443
C	-2.497864	0.161145	0.571734
C	-1.455319	0.344974	1.475164
N	-3.603566	0.318516	-1.441285
C	-4.456100	-0.424285	-0.674197
C	-3.759984	-0.659124	0.695623
C	-3.497663	-2.179145	0.915838
O	-5.578697	-0.815433	-0.944403
O	-4.617590	-0.202037	1.725527
C	-0.300008	1.609173	-0.276252
C	-0.358829	1.074997	1.015862
C	0.813423	3.880760	-0.377609
O	5.722333	-2.087381	-0.372259
O	-0.016228	4.351281	-1.426999
C	-2.476078	-2.753304	0.037697
N	-1.653548	-3.193654	-0.653049
O	3.175948	-2.727227	-0.660299
C	1.822227	-3.145187	-0.823889
H	6.221403	0.326435	0.492043
H	1.397145	-0.744723	-0.021529
H	4.496825	2.041087	1.050141
H	2.265549	2.662140	1.370277
H	1.534012	2.090685	-1.338574
H	-1.292139	1.811649	-2.194879
H	-1.475149	-0.068331	2.478164
H	-3.844863	0.638382	-2.369627
H	-3.190642	-2.313247	1.959093
H	-4.443496	-2.715715	0.771787
H	-5.521913	-0.444598	1.450549
H	0.421816	4.177093	0.608373
H	1.782546	4.372647	-0.512789
H	5.239369	-2.883559	-0.657536
H	-0.921723	4.068362	-1.223775
H	1.301546	-2.529785	-1.565940
H	1.274939	-3.102456	0.124055
H	1.865141	-4.177358	-1.173459

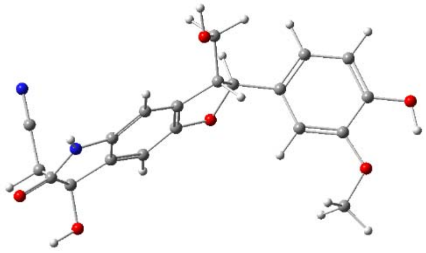

d

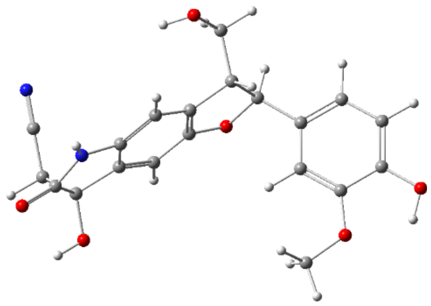

e

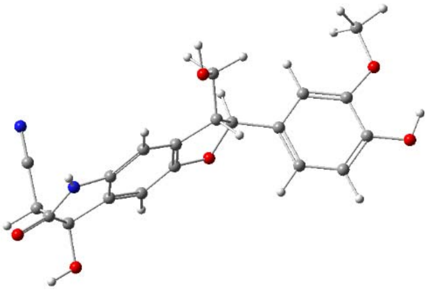

f

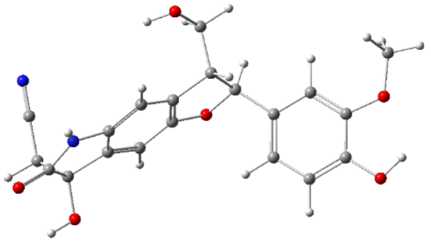

g

Figure S3. Optimized geometries of conformers **d-g** of (3*R*,7'*S*,8'*R*)-**1** at the B3LYP/6-31G(d) level in the gas phase.

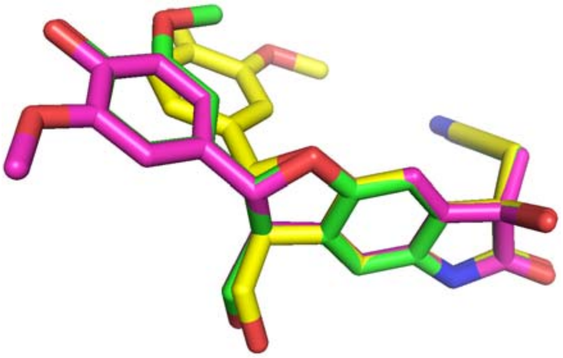

Figure S4. Overlay of conformers **d**–**g**.

Table S5. Important thermodynamic parameters (a.u.) of conformers **d-g** of the optimized (3*R*,7'*S*,8'*R*)-**1** at B3LYP/6-31G(d) level in the gas phase.[a]

species	*E*	*E'*=*E*+*ZPE*	*H*	*G*
**d**	-1333.388187	-1333.413373	-1333.387243	-1333.470294
**e**	-1333.387406	-1333.412524	-1333.386461	-1333.470087
**f**	-1333.386870	-1333.412094	-1333.385926	-1333.469332
**g**	-1333.386485	-1333.411584	-1333.385541	-1333.469018

[a]*E* = total energy; *E'* = total energy with zero point energy (*ZPE*); *H* = enthalpy; *G* = Gibbs free energy.

Table S6. Conformational analysis of conformers **d-g** of (3*R*,7'*S*,8'*R*)-**1**.

species	ΔE^a	$P_E\%^b$	$\Delta E'^c$	$P_{E'}\%^d$	ΔG^e	$P_G\%^f$
**d**	0.00	54.1	0.00	55.1	0.00	41.3
**e**	0.49	23.6	0.53	22.4	0.13	33.1
**f**	0.83	13.4	0.80	14.2	0.60	14.9
**g**	1.07	8.9	1.12	8.3	0.80	10.7

[a]Relative energy, [c]relative energy with *ZPE*, and [e]relative Gibbs free energy in kcal/mol. [b d f] Conformational distribution calculated by using the respective parameters above at B3LYP/6-31G(d) level in the gas phase. (T=298 K)

Table S7. Key transitions, oscillator strengths, and rotatory strengths in the ECD spectra of conformers **d**–**g** at B3LYP-SCRF/6-31+G(d,p)//B3LYP/6-31G(d) level with PCM in MeOH.

species	exited state	$\Delta E\,(eV)^a$	$\lambda\,(nm)^b$	f^c	$R_{vel}{}^d$	$R_{len}{}^e$
**d**	100->101	3.9439	314.37	0.0329	5.0759	4.6844
	99->101	4.3621	284.23	0.0017	-0.5467	-0.5452
	100->102	4.5334	273.49	0.2391	-8.5892	-8.6146
	100->103	4.7835	259.19	0.0345	-18.3916	-17.2732
	99->103	4.8631	254.95	0.0551	2.6652	2.6433
	99->102	4.9273	251.63	0.0058	7.1579	7.4224
	100->104	5.0324	246.37	0.0012	1.6544	1.4623
	98->101	5.1405	241.19	0.0032	7.9308	8.4134
	100->105	5.1774	239.47	0.0278	-5.7401	-5.6978
	96->101	5.2230	237.38	0.0173	-56.8127	-57.6183
	100->106	5.2444	236.41	0.0015	-2.9557	-3.7109
	100->106	5.2649	235.49	0.0005	1.4857	1.7530
	100->108	5.3890	230.07	0.0266	2.2142	2.5020
	99->105	5.4426	227.80	0.1572	-17.5775	-17.8795
	100->109	5.4755	226.43	0.1282	33.2521	34.2324
	100->110	5.5227	224.50	0.0053	6.6077	7.2290
	99->104	5.5262	224.36	0.0002	-0.7546	-0.5745
	97->101	5.5868	221.92	0.3380	-21.4339	-22.7327
	100->107	5.6484	219.50	0.0281	0.4981	0.6363
	99->106	5.6923	217.81	0.0024	-4.3668	-4.0974
	98->102	5.7036	217.38	0.0188	7.3609	7.0130
	100->111	5.7120	217.06	0.0224	-3.7859	-3.5692
	99->108	5.7693	214.90	0.0179	11.0500	10.3767
	100->112	5.8049	213.58	0.0221	-26.7309	-26.7413
	100->113	5.8442	212.15	0.0053	10.5408	10.1129
	99->108	5.8652	211.39	0.0041	-6.9570	-7.7079
	100->113	5.8844	210.70	0.0293	12.5867	12.6936
	99->111	5.9413	208.68	0.0138	12.6766	13.8595
	95->101	5.9579	208.10	0.0023	3.8070	3.3216
	100->114	5.9980	206.71	0.0208	4.4190	4.3776
**e**	99->101	4.2353	292.74	0.0112	-2.2127	-1.9135
	100->102	4.4796	276.77	0.2647	-42.1484	-43.0312
	99->102	4.7366	261.76	0.0069	1.1515	1.3427
	100->103	4.8444	255.93	0.0275	9.8079	10.5402
	99->103	4.8843	253.84	0.0304	-12.8636	-13.2252
	100->104	5.0063	247.66	0.0016	5.7228	5.5101
	98->101	5.0537	245.34	0.0039	0.1903	0.5579
	100->105	5.1646	240.07	0.0287	16.1772	16.8898
	99->107	5.2134	237.82	0.0040	-12.9114	-14.3292
	96->101	5.2155	237.72	0.0148	-47.2625	-47.4170
	100->106	5.2255	237.27	0.0009	0.2402	-0.2866

	99->104	5.3924	229.92	0.0983	-40.4147	-41.1573
	100->108	5.3999	229.60	0.0106	0.4030	0.1661
	99->105	5.4404	227.90	0.0887	27.3304	28.2470
	100->107	5.4919	225.76	0.0978	16.7562	18.2121
	98->102	5.5377	223.89	0.1240	-25.7290	-26.7257
	100->110	5.5602	222.98	0.0303	-2.6844	-3.2316
	98->102	5.6087	221.06	0.1246	32.8275	32.1538
	100->110	5.6191	220.65	0.0402	3.5078	4.0612
	99->106	5.6509	219.41	0.0009	1.7658	2.0338
	99->108	5.6931	217.78	0.0038	5.6435	3.8199
	100->111	5.7428	215.89	0.0121	-2.2859	-2.4562
	99->108	5.7702	214.87	0.0020	-0.6571	-0.6410
	100->112	5.8458	212.09	0.0530	-33.1359	-33.3945
	99->111	5.8881	210.57	0.0129	15.8336	15.2620
	100->113	5.9490	208.41	0.0286	-1.1345	-2.2229
	95->101	5.9606	208.01	0.0299	26.5756	26.2698
	99->109	5.9637	207.90	0.0116	-15.4563	-14.0367
	99->112	6.0262	205.74	0.0133	22.0536	23.3555
	100->101	3.9382	314.82	0.0339	5.4058	5.3483
	99->101	4.3551	284.69	0.0004	0.8635	0.8687
	100->102	4.5276	273.84	0.2598	0.7774	0.5653
	100->103	4.8448	255.91	0.0437	-2.5758	-2.8962
	99->102	4.9013	252.96	0.0063	-7.5643	-7.1991
	100->104	4.9209	251.96	0.0116	-6.3406	-5.9723
	100->105	5.0146	247.25	0.0071	-0.5209	-0.3413
	100->105	5.0355	246.22	0.0038	1.2856	0.8565
	98->101	5.1997	238.44	0.0172	-29.3333	-29.5914
	99->107	5.2208	237.48	0.0002	-1.0594	-0.7306
	96->101	5.2348	236.85	0.0066	-24.9112	-25.2866
	100->106	5.2529	236.03	0.0027	7.5221	8.4724
	99->104	5.3373	232.30	0.1629	-9.7452	-10.7904
**f**	100->108	5.3835	230.31	0.0195	1.8059	2.0016
	100->109	5.4543	227.31	0.0279	13.2476	13.0766
	99->105	5.5062	225.17	0.0145	2.4312	3.0574
	100->110	5.5305	224.18	0.0135	1.4050	1.3884
	97->101	5.5897	221.81	0.4992	52.9020	54.3936
	99->106	5.6343	220.05	0.0247	-5.3481	-5.5456
	99->109	5.6613	219.00	0.0035	-10.2846	-10.8107
	100->112	5.7264	216.51	0.0021	1.2366	1.7023
	100->112	5.7445	215.83	0.0189	-10.1397	-10.5501
	98->102	5.7641	215.10	0.0097	-8.1921	-8.4692
	100->111	5.7969	213.88	0.0178	-5.1125	-5.0131
	99->108	5.8439	212.16	0.0136	2.1893	2.4207
	100->113	5.8563	211.71	0.0038	10.6240	10.5067
	99->109	5.8852	210.67	0.0015	-0.1082	0.1430

	99->112	5.9370	208.83	0.0040	0.4272	0.4775
	95->101	5.9586	208.08	0.0145	14.2329	13.4319
	100->114	5.9683	207.74	0.0457	1.8061	2.0449
**g**	100->101	3.9245	315.92	0.0301	7.4489	7.8808
	99->101	4.2413	292.33	0.0013	-4.0182	-4.3348
	100->102	4.4673	277.54	0.2857	-56.9799	-56.1161
	99->102	4.7214	262.60	0.0129	21.9884	22.2168
	100->103	4.8411	256.11	0.0275	10.9408	11.9496
	99->103	4.9796	248.98	0.0124	-5.3947	-5.7168
	100->104	5.0351	246.24	0.0007	-0.9002	-1.1190
	98->101	5.0573	245.16	0.0574	-13.8487	-13.5859
	100->105	5.1707	239.78	0.0078	11.7601	11.0296
	99->107	5.1987	238.49	0.0008	-5.8275	-4.5048
	96->101	5.2173	237.64	0.0140	-47.5117	-47.3272
	100->106	5.2275	237.18	0.0014	6.9954	7.1280
	100->108	5.3994	229.62	0.0156	-14.1670	-14.2621
	99->104	5.4335	228.19	0.0236	18.8711	17.6141
	99->105	5.4540	227.33	0.0803	-28.6633	-25.6407
	100->109	5.5035	225.28	0.0458	24.4253	23.3259
	100->110	5.5736	222.45	0.0321	14.3910	13.3462
	98->102	5.5773	222.30	0.0889	-17.1620	-16.4248
	97->101	5.5961	221.56	0.2506	29.1225	29.1515
	99->106	5.6293	220.25	0.0074	-4.4708	-3.1266
	100->107	5.6570	219.17	0.0404	-4.7506	-4.7770
	99->106	5.6853	218.08	0.0012	-0.2243	-0.0766
	99->108	5.7555	215.42	0.0035	-2.1737	-2.3295
	100->112	5.7848	214.33	0.0019	0.8941	0.9268
	100->112	5.8419	212.23	0.0100	-6.5587	-6.7474
	100->113	5.9163	209.56	0.0662	-30.6948	-29.8491
	99->110	5.9308	209.05	0.0183	-8.2913	-8.2859
	95->101	5.9594	208.05	0.0459	12.3440	12.5501
	99->112	5.9849	207.16	0.0013	1.4283	1.3606
	99->111	6.0291	205.64	0.0034	1.0446	0.7916

Table S8. Optimized Z-matrixes of conformers **d-g** of (3*R*,7'*S*,8'*R*)-**1** in the gas phase (Å) at B3LYP/6-31G(d) level.

**d**				**e**			
C	-5.298181	1.001133	1.028878	C	5.367831	0.799221	-0.258714
C	-5.651942	-0.174123	0.379115	C	5.359823	-0.550724	0.069154
C	-4.640647	-1.024521	-0.120630	C	4.138246	-1.257984	0.068947
C	-3.302005	-0.685850	0.022595	C	2.949034	-0.612294	-0.246215
C	-2.945599	0.505988	0.678648	C	2.958985	0.754363	-0.572822
C	-3.948126	1.333438	1.183029	C	4.170967	1.444694	-0.583887
C	-1.497927	0.906940	0.789567	C	1.670497	1.492754	-0.855197
C	-0.851030	1.474011	-0.514714	C	0.902759	1.983804	0.421535
O	-0.680173	-0.237223	1.170755	O	0.738123	0.641953	-1.590906
C	1.682423	1.132132	-1.201546	C	-1.361967	0.977158	1.333513
C	2.800803	0.357545	-0.907177	C	-2.405520	0.122790	0.986369
C	2.791790	-0.629858	0.090223	C	-2.440800	-0.554134	-0.242738
C	1.648853	-0.883696	0.841753	C	-1.412384	-0.421040	-1.169883
N	4.078798	0.401514	-1.514584	N	-3.549639	-0.216469	1.745810
C	4.920149	-0.542908	-0.999391	C	-4.337981	-1.126393	1.098002
C	4.170583	-1.241175	0.169749	C	-3.723700	-1.348488	-0.312403
C	4.907813	-0.976139	1.514189	C	-4.712839	-0.854790	-1.408333
O	6.038188	-0.854023	-1.374731	O	-5.320651	-1.714986	1.514674
O	4.160331	-2.639411	-0.063015	O	-3.497603	-2.733591	-0.503997
C	0.537479	0.879794	-0.445588	C	-0.334492	1.121831	0.397769
C	0.529760	-0.107689	0.543006	C	-0.361228	0.424781	-0.812689
C	-0.921886	2.996401	-0.580894	C	0.578135	3.492343	0.340274
O	-6.961845	-0.513632	0.232228	O	6.520329	-1.192238	0.378042
O	-0.426452	3.399392	-1.854411	O	-0.217946	3.943763	1.423185
C	4.955478	0.431729	1.913559	C	-4.987493	0.582771	-1.366577
N	4.994116	1.551179	2.217614	N	-5.197120	1.723176	-1.315429
O	-5.128442	-2.158539	-0.723267	O	4.274524	-2.585483	0.396391
C	-4.196622	-3.097758	-1.241199	C	3.110616	-3.400014	0.399958
H	-6.085244	1.638173	1.419540	H	6.315631	1.327959	-0.268007
H	-2.521032	-1.342314	-0.343457	H	2.013519	-1.158623	-0.261849
H	-3.682114	2.246363	1.710038	H	4.191584	2.498724	-0.850547
H	-1.389147	1.652764	1.590906	H	1.885622	2.342873	-1.512985
H	-1.384660	1.085253	-1.393509	H	1.500429	1.814397	1.323420
H	1.683639	1.899487	-1.967920	H	-1.330800	1.491950	2.289160
H	1.617771	-1.644088	1.615110	H	-1.410243	-0.953039	-2.115495
H	4.319064	0.963222	-2.320097	H	-3.715317	0.061216	2.703863
H	4.408382	-1.561714	2.293832	H	-4.294702	-1.121157	-2.385238
H	5.933772	-1.351822	1.416010	H	-5.655605	-1.401754	-1.284450
H	5.033390	-2.852135	-0.443405	H	-4.258929	-3.187518	-0.095893
H	-1.967656	3.316378	-0.445209	H	1.512342	4.062734	0.387799
H	-0.324622	3.429117	0.236949	H	0.100735	3.713199	-0.627021
H	-6.982060	-1.369683	-0.230910	H	6.290258	-2.120280	0.560885
H	-0.414410	4.368171	-1.878158	H	-1.128240	3.657361	1.250143
H	-3.543111	-3.485331	-0.449818	H	2.379073	-3.044381	1.136486
H	-3.582420	-2.651679	-2.033572	H	3.444211	-4.402378	0.673568

H	-4.789791	-3.913898	-1.657042	H	2.642611	-3.427460	-0.591984
		**f**				**g**	
C	-4.236408	-1.771650	-1.267916	C	3.568023	-2.327048	0.032065
C	-5.370535	-1.257654	-0.646117	C	4.828145	-1.784361	0.250006
C	-5.229480	-0.269646	0.348275	C	5.032007	-0.403142	0.047703
C	-3.964909	0.189170	0.705191	C	3.980123	0.407242	-0.363399
C	-2.822365	-0.321118	0.068384	C	2.703104	-0.143251	-0.571831
C	-2.970619	-1.303460	-0.914089	C	2.509439	-1.509539	-0.376164
C	-1.462899	0.219786	0.429287	C	1.571023	0.785806	-0.960633
C	-0.777094	1.112772	-0.653332	C	0.892868	1.517274	0.248955
O	-0.535176	-0.873454	0.687480	O	0.510398	0.055316	-1.639480
C	1.832151	1.210966	-1.083327	C	-1.461587	0.896402	1.272376
C	3.012815	0.527420	-0.808369	C	-2.619206	0.169741	1.004870
C	3.044667	-0.634578	-0.021760	C	-2.772909	-0.587812	-0.166683
C	1.881184	-1.166028	0.525228	C	-1.755539	-0.672168	-1.111574
N	4.324544	0.840698	-1.239403	N	-3.783327	0.052992	1.799644
C	5.234629	-0.084093	-0.813342	C	-4.702765	-0.785710	1.233273
C	4.480963	-1.083697	0.107932	C	-4.155375	-1.196560	-0.162260
C	5.044707	-1.004563	1.556562	C	-5.087887	-0.648048	-1.282179
O	6.415221	-0.182095	-1.103940	O	-5.748190	-1.198215	1.704901
O	4.687121	-2.399300	-0.376676	O	-4.130849	-2.609097	-0.252638
C	0.666259	0.680571	-0.530660	C	-0.443767	0.822135	0.318254
C	0.699452	-0.480745	0.245610	C	-0.591411	0.043959	-0.832044
C	-1.047581	2.597865	-0.432146	C	0.749416	3.033855	-0.009978
O	-6.606888	-1.711747	-0.990005	O	5.862104	-2.577607	0.646405
O	-0.501336	3.309289	-1.538411	O	0.031147	3.701785	1.013052
C	4.851188	0.289010	2.214675	C	-5.153946	0.813155	-1.348504
N	4.698837	1.319895	2.725803	N	-5.197808	1.972468	-1.383813
O	-6.424532	0.144161	0.888470	O	6.323865	0.003068	0.284188
C	-6.394312	1.103726	1.933822	C	6.642916	1.373643	0.099567
H	-4.362906	-2.540195	-2.023897	H	3.428857	-3.393028	0.182409
H	-3.853242	0.937139	1.483698	H	4.141548	1.468211	-0.529943
H	-2.090906	-1.720822	-1.394270	H	1.534001	-1.946915	-0.556579
H	-1.536643	0.794599	1.365071	H	1.940690	1.520780	-1.686247
H	-1.165783	0.855842	-1.648545	H	1.476832	1.379845	1.164827
H	1.801211	2.111562	-1.686859	H	-1.340884	1.476648	2.182384
H	1.880870	-2.065961	1.131198	H	-1.846851	-1.271695	-2.011193
H	4.559724	1.573543	-1.894989	H	-3.886055	0.417686	2.737110
H	4.565454	-1.792327	2.148111	H	-4.732952	-1.043707	-2.240087
H	6.118785	-1.222782	1.511791	H	-6.095968	-1.043193	-1.106537
H	5.611893	-2.427452	-0.686759	H	-4.934132	-2.921482	0.204803
H	-2.135266	2.760891	-0.356533	H	1.745045	3.491532	-0.032050
H	-0.588905	2.917903	0.516582	H	0.286930	3.194676	-0.996249
H	-7.248936	-1.235497	-0.434664	H	6.643523	-2.003807	0.732097
H	-0.606495	4.257381	-1.367387	H	-0.907584	3.490049	0.890621
H	-5.951040	2.048772	1.594217	H	6.055808	2.012534	0.771776
H	-7.433857	1.273669	2.219229	H	7.703055	1.473030	0.338652
H	-5.833771	0.730432	2.800401	H	6.472237	1.685180	-0.938945

Molecular Orbital (MO) Analysis of Plasiatine (1)

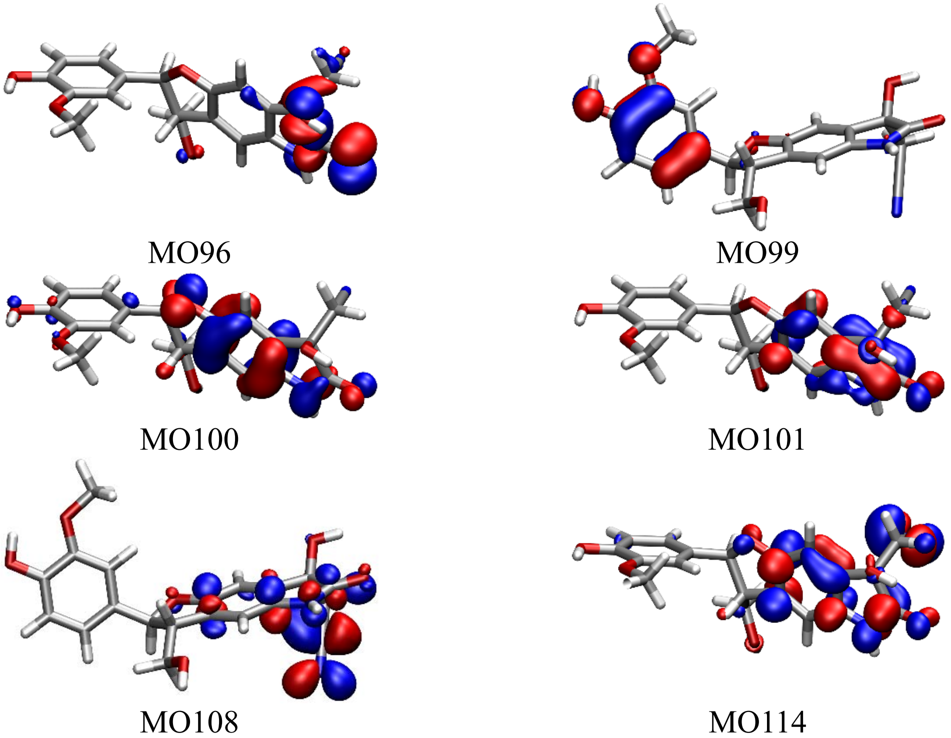

Figure S5. Important MOs in the ECD spectrum of the predominant conformer **d** at B3LYP/6-31+G(d,p) level with PCM in MeOH.

NMR, MS, IR, UV, and ECD Spectra of Plasiatine (1)

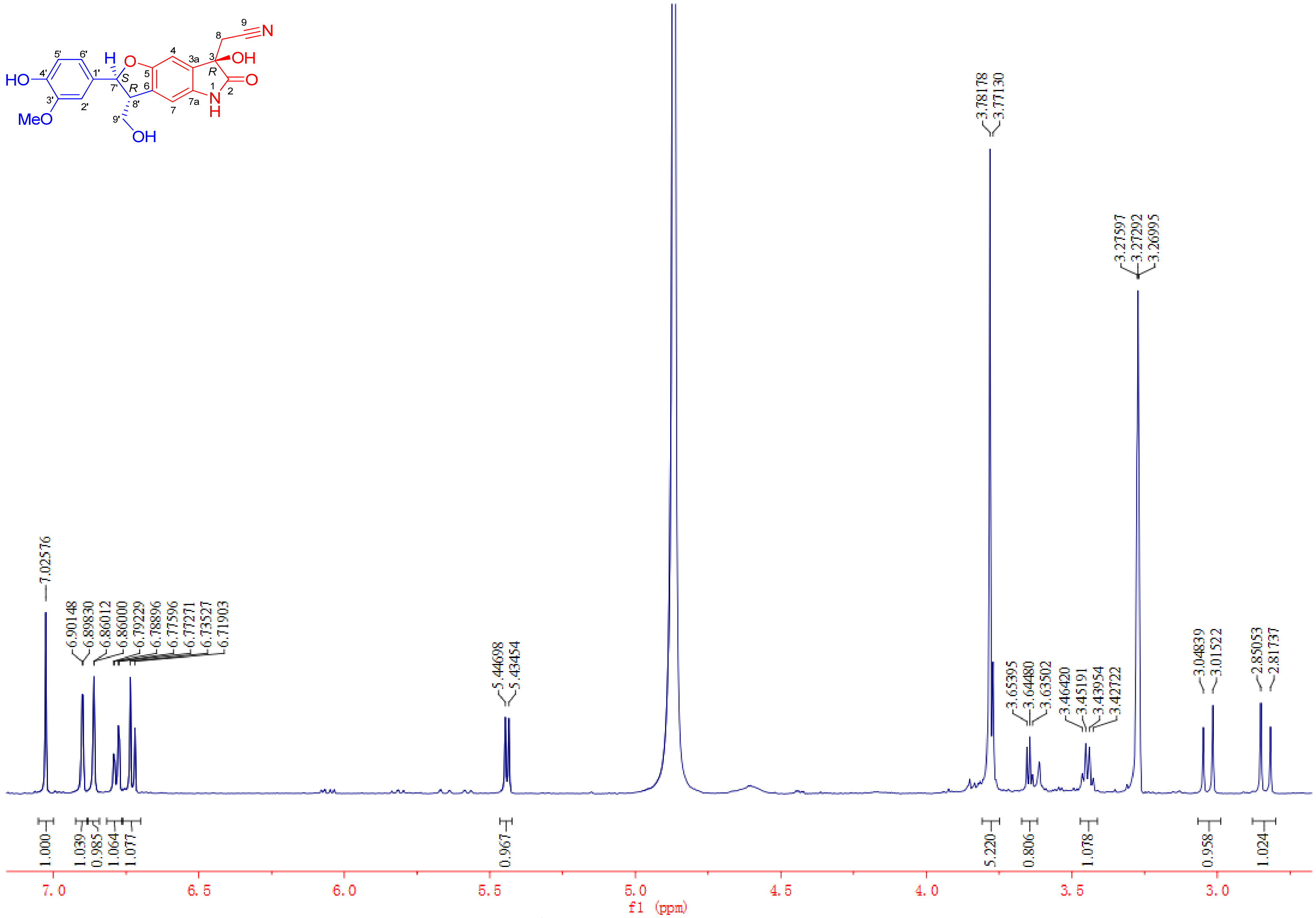

Figure S6. ^{1}H NMR spectrum of plasiatine (**1**).

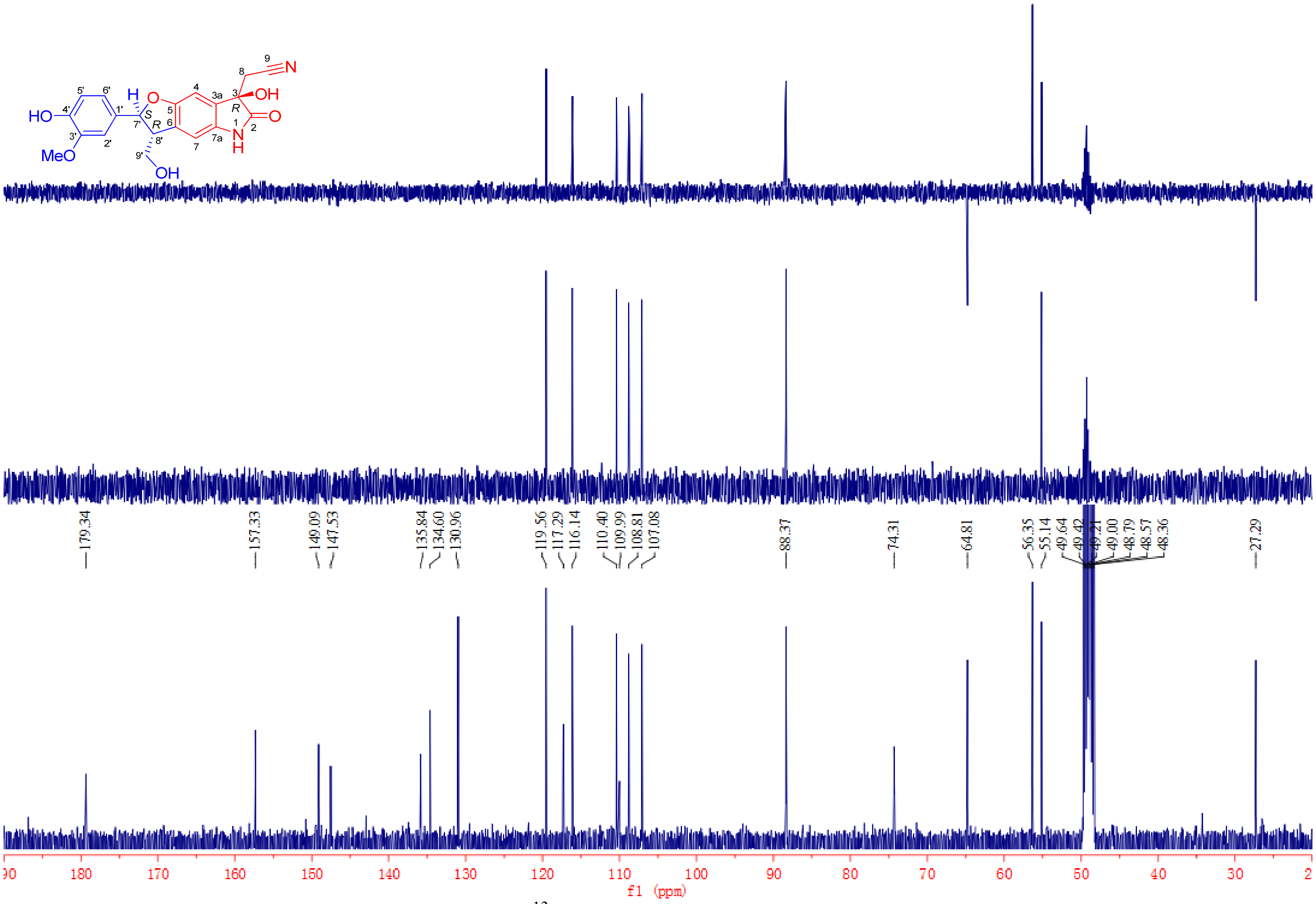

Figure S7. ^{13}C NMR spectrum of plasiatine (**1**).

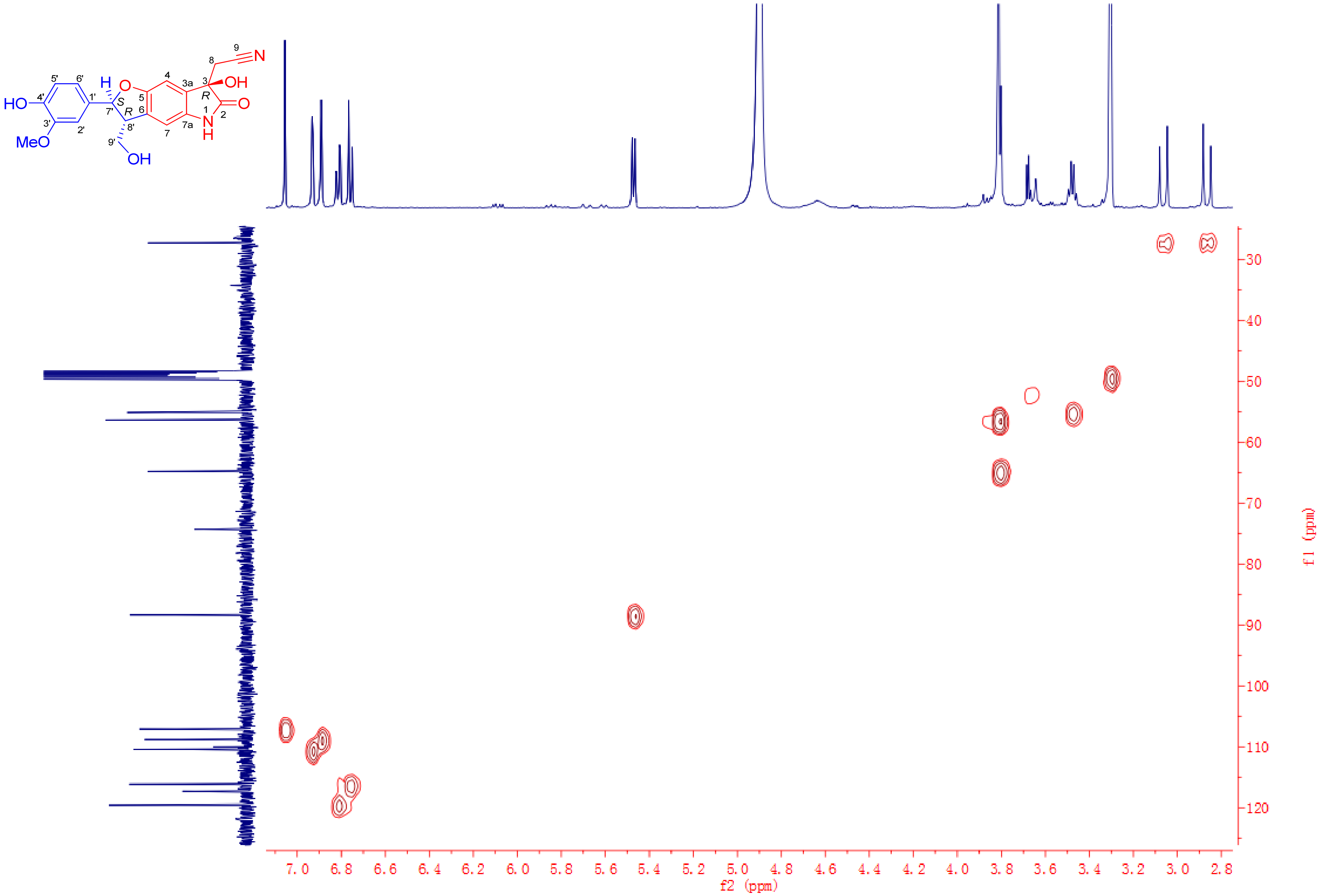

Figure S8. HSQC spectrum of plasiatine (**1**).

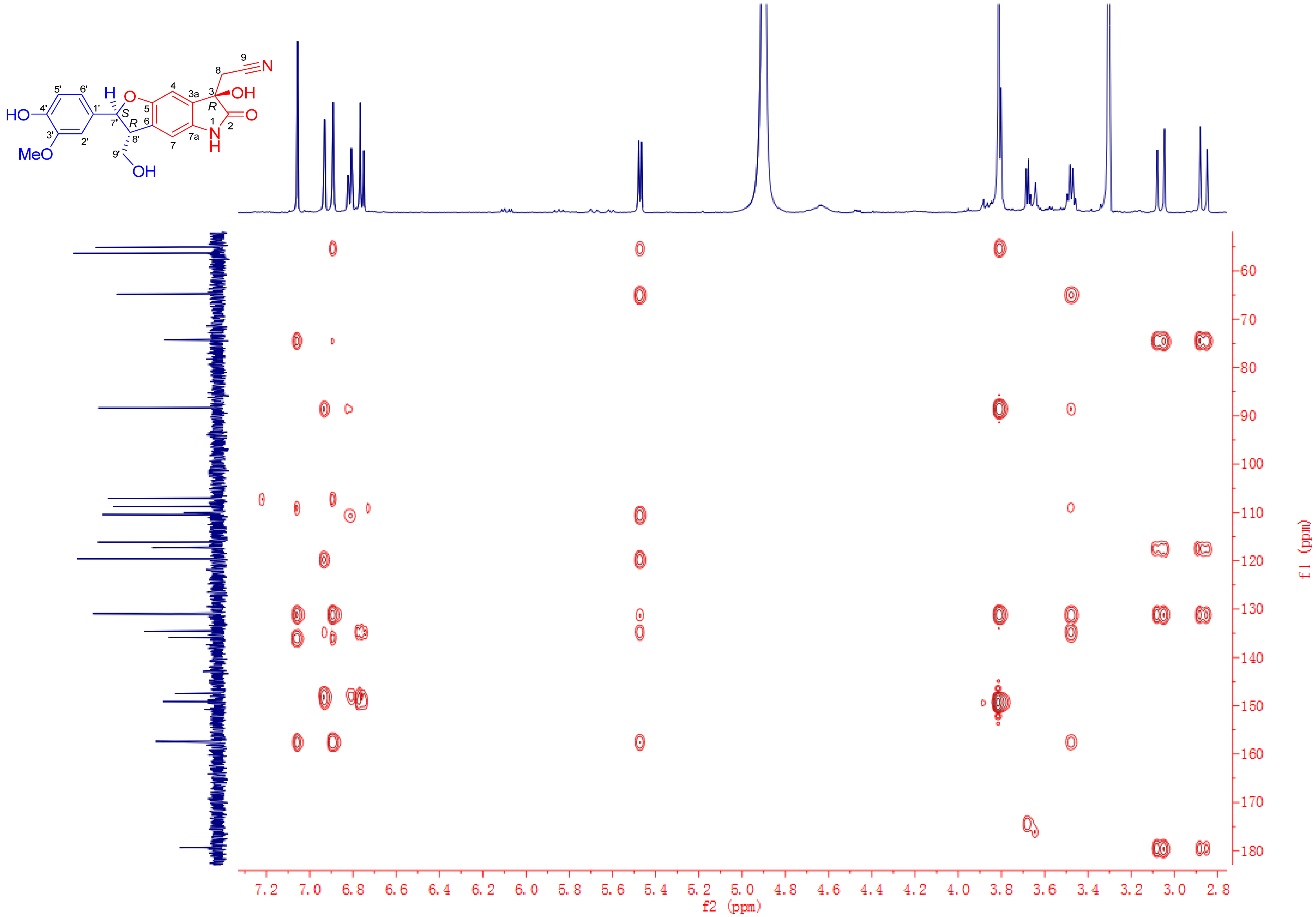

Figure S9. HMBC spectrum of plasiatine (**1**).

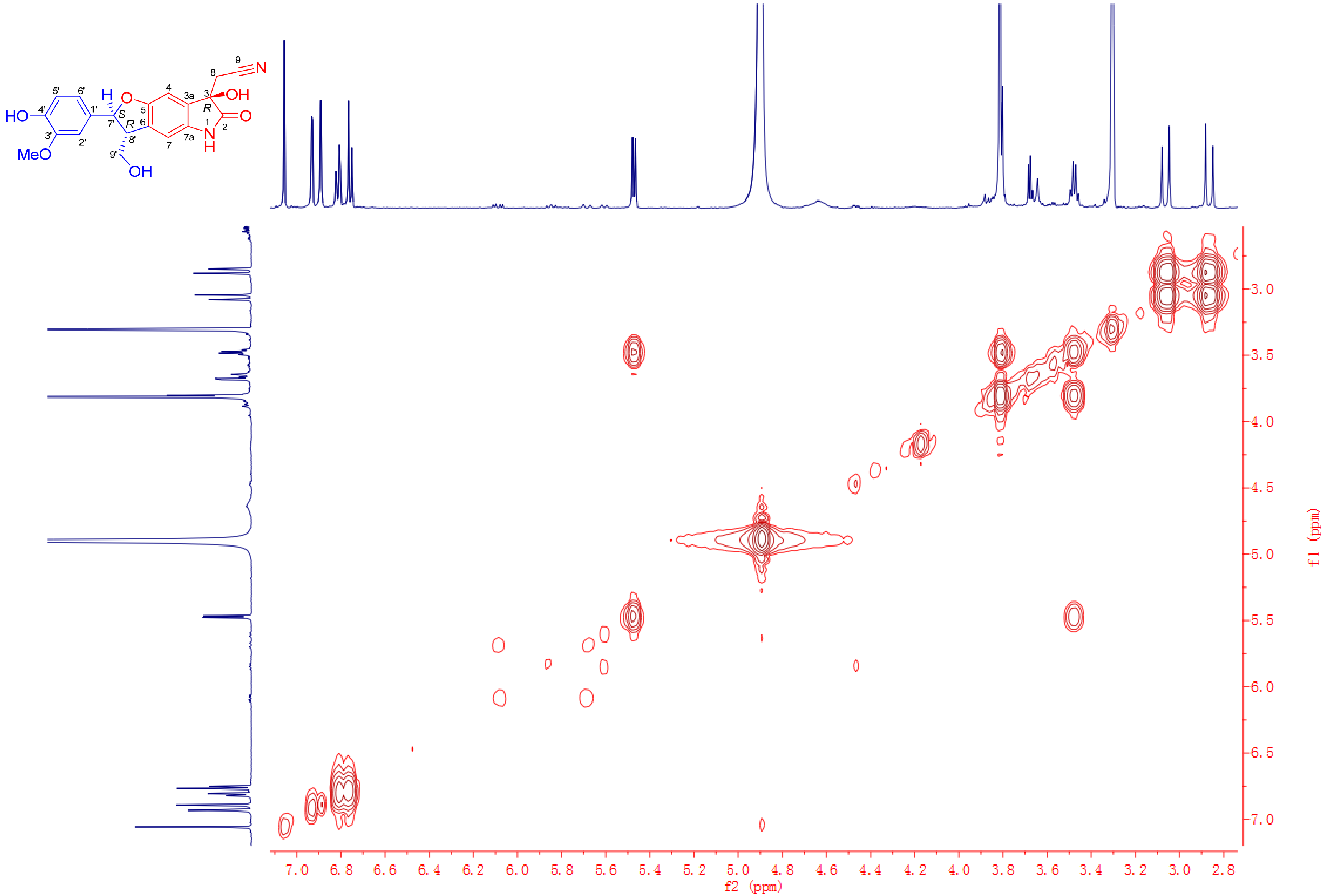

Figure S10. ^{1}H–^{1}H COSY spectrum of plasiatine (**1**).

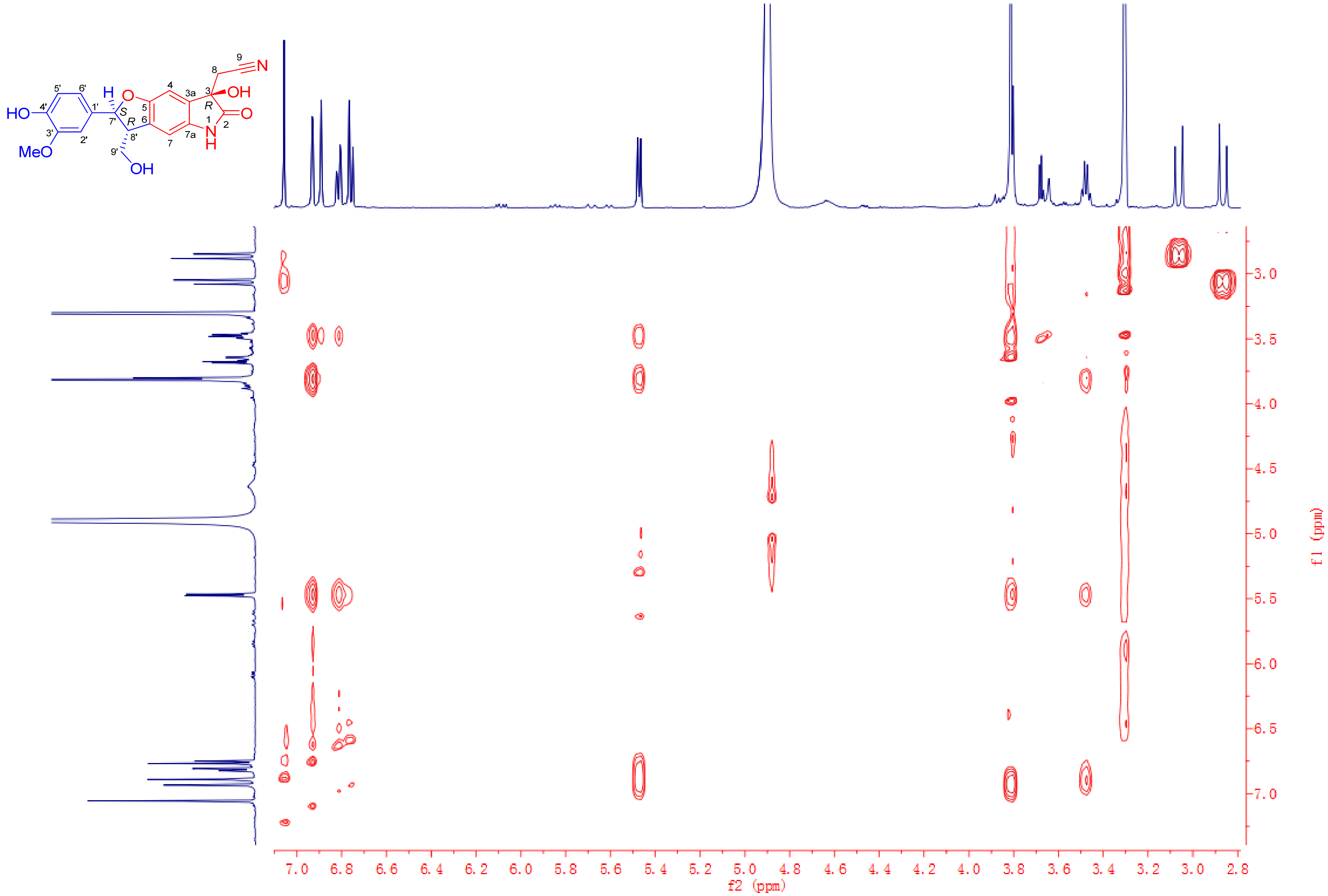

Figure S11. ROESY spectrum of plasiatine (**1**).

Elemental Composition Report **Page 1**

Single Mass Analysis
Tolerance = 10.0 PPM / DBE: min = -10.0, max = 120.0
Selected filters: None

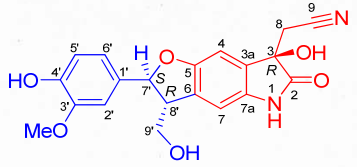

Monoisotopic Mass, Odd and Even Electron Ions
21 formula(e) evaluated with 1 results within limits (up to 51 closest results for each mass)
Elements Used:
C: 0-200 H: 0-400 N: 2-2 O: 4-7

sky-58b
16:32:18 06-Jul-2012
Voltage EI+

KIB
M120706EA-10AFAMM 27 (2.479)

Autospec Premier
P776
49.1

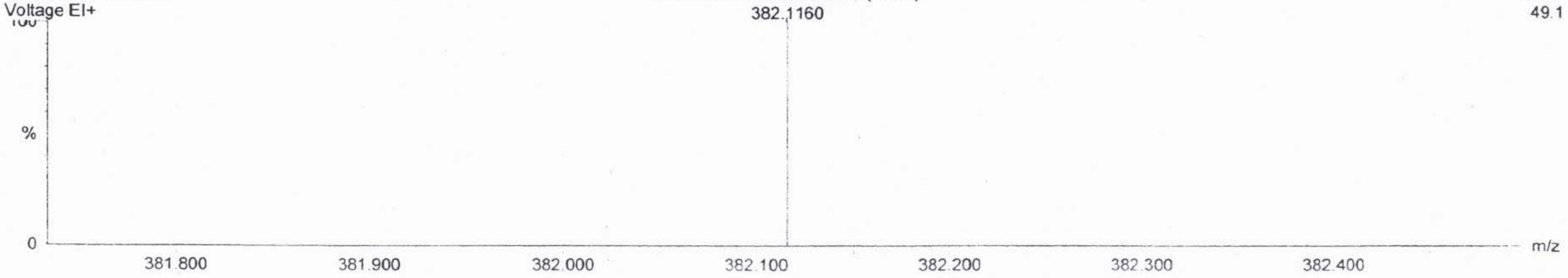

Minimum:				-10.0		
Maximum:		100.0	10.0	120.0		
Mass	Calc. Mass	mDa	PPM	DBE	i-FIT	Formula
382.1160	382.1165	-0.5	-1.3	13.0	5546040.0	C20 H18 N2 O6

Figure S12. HREIMS spectrum of plasiatine (**1**).

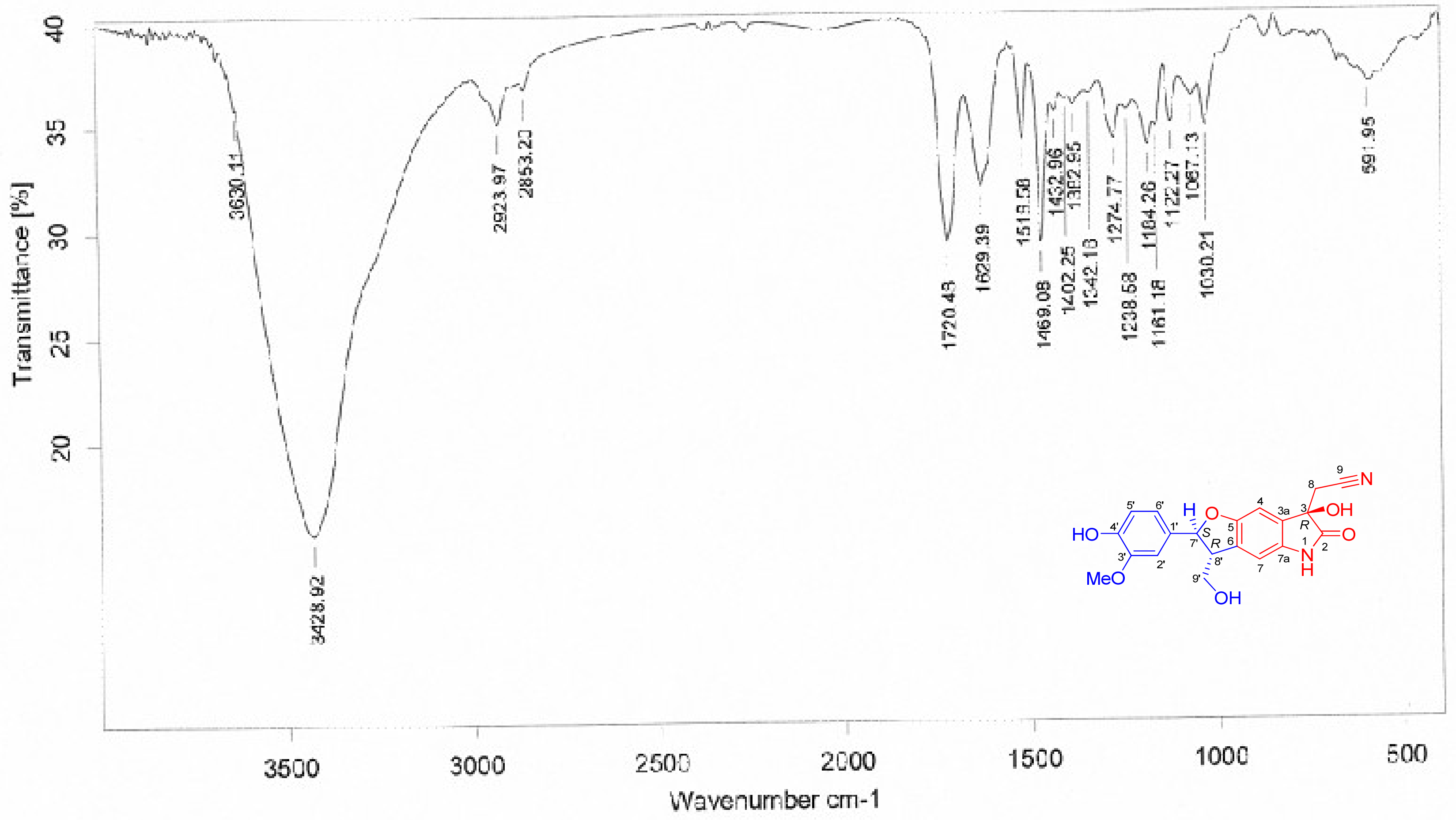

Sample : sky-58b		Frequency Range : 399.246 - 3996.32	Measured on : 31/12/2002
Technique : KBr压片	Resolution : 4	Instrument : Tensor27	Sample Scans : 16
Customer : 121018IR4	Zerofilling : 2	Acquisition : Double Sided,For	

Figure S13. IR spectrum of plasiatine (**1**).

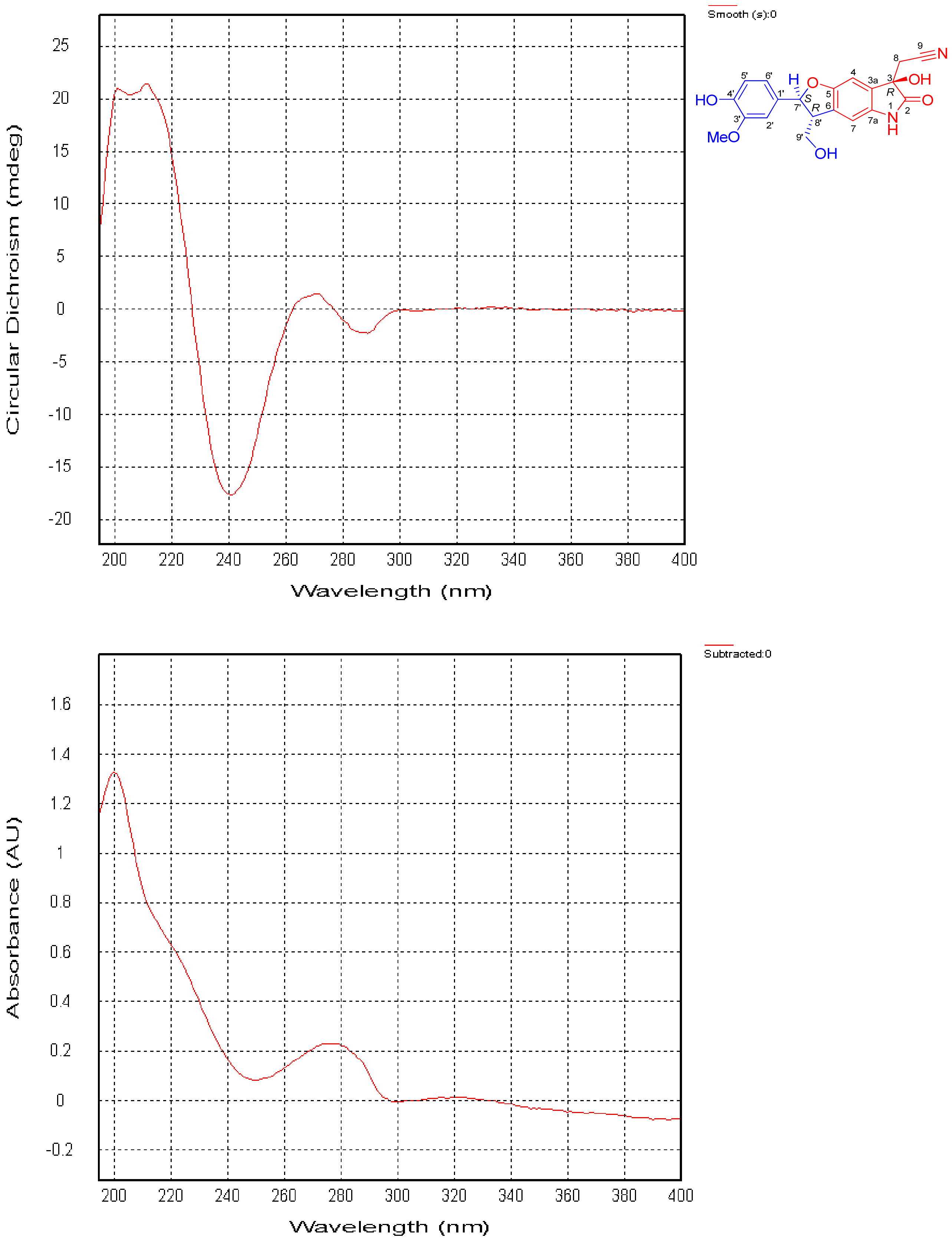

Figure S14. ECD (top) and UV (bottom) spectra of plasiatine (**1**).
